# Differential Role for a Defined Lateral Horn Neuron Subset in Naïve Odor Valence in *Drosophila*

**DOI:** 10.1038/s41598-020-63169-3

**Published:** 2020-04-09

**Authors:** Hadas Lerner, Eyal Rozenfeld, Bar Rozenman, Wolf Huetteroth, Moshe Parnas

**Affiliations:** 1 Department of Physiology and Pharmacology, Sackler School of Medicine, Tel Aviv University, Tel Aviv, 69978 Israel; 2 Sagol School of Neuroscience, Tel Aviv University, Tel Aviv, 69978 Israel; 3 Institute for Biology, University of Leipzig, Talstraße 33, 04103 Leipzig, Germany

**Keywords:** Neural circuits, Olfactory system

## Abstract

Value coding of external stimuli in general, and odor valence in particular, is crucial for survival. In flies, odor valence is thought to be coded by two types of neurons: mushroom body output neurons (MBONs) and lateral horn (LH) neurons. MBONs are classified as neurons that promote either attraction or aversion, but not both, and they are dynamically activated by upstream neurons. This dynamic activation updates the valence values. In contrast, LH neurons receive scaled, but non-dynamic, input from their upstream neurons. It remains unclear how such a non-dynamic system generates differential valence values. Recently, PD2a1/b1 LH neurons were demonstrated to promote approach behavior at low odor concentration in starved flies. Here, we demonstrate that at high odor concentrations, these same neurons contribute to avoidance in satiated flies. The contribution of PD2a1/b1 LH neurons to aversion is context dependent. It is diminished in starved flies, although PD2a1/b1 neural activity remains unchanged, and at lower odor concentration. In addition, PD2a1/b1 aversive effect develops over time. Thus, our results indicate that, even though PD2a1/b1 LH neurons transmit hard-wired output, their effect on valence can change. Taken together, we suggest that the valence model described for MBONs does not hold for LH neurons.

## Introduction

In order to survive, animals must attach value to external stimuli, be it due to innate or learned behavior. In particular, the ability to accurately evaluate potential food resources is a critical trait for survival. To make such an assessment, animals use many senses, with olfaction often serving as a primary cue.

The *Drosophila* olfactory system resembles that of mammals, including our own, and uses similar principles to decode olfactory information^1,2^. Odors bind to olfactory receptor neurons (ORNs), which are located in the antennae and maxillary palps, where each ORN expresses a single type of odorant receptor (OR)^3–5^. All ORNs expressing the same OR converge onto the same region in the antennal lobe termed the glomerulus^6–8^. Second-order excitatory cholinergic projection neurons (ePNs) have dendrites that are restricted to a single glomerulus, whereas inhibitory GABAergic projection neurons (iPNs) are mostly multiglomerular^9^. Both PN types project to the lateral horn (LH), whereas only ePNs project to the calyx of the mushroom body (MB)^9^. Until recently, associative learning and memory processes were generally believed to occur in the MB, with innate behavior driven by the LH^10,11^. However, although the LH is still believed to contribute greatly to innate behavior, it has become apparent that the rigid functional distinction between the two neuropils cannot be upheld. There is now evidence that the MB also plays a role in some innate olfactory behaviors, mostly attractive^12–14^, while the LH is involved in some forms of associative memory^15^.

The LH compartment contains over 1300 cells that are categorized into over 150 types, each with individual morphology^16^. Cells that share morphological features are also more likely to share PN connectivity, although there is some variability^17^. Nine LH cell types could be distinguished by optogenetic activation to drive either attraction (3 cell types) or aversion (6 cell types)^18^. In the case of odor stimuli, effects on odor valence were demonstrated for only three types of LH neurons and under very specific conditions: I. AV1a1 LH neurons, which trigger aversion and are required for geosmin avoidance^19^ II. LH neurons, labeled by the R21G11- and R23C09-GAL4 driver lines, and which process CO_2_ avoidance^20^. III. PD2a1/b1 neurons (previously known as type I LH neurons^21^ or ML9 and ML8^17^, respectively).

PD2a1/b1 neurons belong to the lateral horn output neurons (LHON)^15^. They have their somata in the lateral posteriodorsal protocerebrum, extend a short primary neurite towards the brain center and then bifurcate to connect their input regions in the LH (PD2a1/b1) and in the MB (PD2b1 only) with their presynaptic target areas in the superior intermediate protocerebrum (SIP) and superior medial protocerebrum (SMP) around the vertical MB stalk^15^. About one third of input synapses in both LH and calyx derive from uniglomerular PNs, with another third provided by local LH neurons. In addition, reciprocal LHON input accounts for about 20%, and a varying amount of ipsi- and contralateral axoaxonic input in the SIP comes from the mushroom body output neuron (MBON)-α2sc^15^. PD2a1/b1 neurons were found to contribute to food odor approach at odor concentrations in the range of 10^−7^ to 10^−5^ dilution in starved flies^15^. In addition, PD2a1/b1 neurons were also shown to be required for aversive conditioning and it was suggested that reduced activation of PD2a1/b1 neurons following aversive conditioning was responsible for the reduced odor approach^15^. These observations are in agreement with current knowledge about learning and memory processes occurring at the MB and MBONs. Accordingly, MBONs are divided into neurons that drive either attraction or aversion, and plasticity between MB and MBONs shifts the balance between attraction and aversion for each odor^22–30^. However, in contrast to the known plasticity of the synapse between MB neurons and MBONs, there is no information about any such similar plasticity between PNs and LH neurons. Furthermore, optogenetic activation of PD2a1/b1 neurons generated a mild aversion^18^ rather than attraction^15^. In this context, odor valence can range from attractive to aversive, depending on the interplay of external factors (such as odor concentration) and internal states (such as satiation level)^31^, where a high odor concentration (even in the case of food odors) is usually associated with aversion^14,32–35^. PN input to PD2a1/b1 neurons is positively correlated with odor concentration and as a result the activity of PD2a1/b1 neurons increases linearly with odor concentration^21^. Thus, it is unclear how increased input to neurons which underlie attraction supports an overall aversive response to odors. This seemingly contradicts the notion that PD2a1/b1 neurons only drive attraction. We therefore examined whether PD2a1/b1 neurons always contribute to approach behavior or whether their role is context dependent. Thus, we examined the contribution of PD2a1/b1 neurons to behavior responses under broader conditions and in particular, higher odor concentrations and satiated flies.

Here we show that PD2a1/b1 neurons, which were found to mediate an approach to odors in starved flies and at low odor concentrations, can also contribute to odor avoidance at higher odor concentrations in satiated flies. In accordance with these observations, the effects we saw on avoidance behavior are influenced not only by odor identity (there are differences even between odors with similar activation patterns) and odor concentration, but also by exposure time, and satiation. In particular, starvation abolished the contribution of PD2a1/b1 neurons to an aversive response to odors even though there was no effect on the PD2a1/b1 neuron odor responses.

## Results

### PD2a1/b1 neurons exhibit a spectrum of odor responses

Selecting suitable odors for behavioral experiments requires prior knowledge about the physiological responses of PD2a1/b1 neurons. We therefore used two GAL4 driver lines to characterize the response profile of PD2a1/b1 neurons. The first one, R37G11-GAL4, labels 6–7 PD2a1/b1 neurons and is a relatively clean line that is also suitable for behavioral experiments^15^ (Supplementary Fig. S1), while R48F03-GAL4 labels 18–22 PD2a1/b1 neurons and allows a larger coverage of the PD2a1/b1 population. Unfortunately, the R48F03-GAL4 driver line was also strongly expressed in additional neurons (e.g., in the subesophageal ganglion, the fan-shaped body, and the thoracico-abdominal ganglia). This line was therefore not suitable for all the characterizations and specifically could not be used for the behavioral experiments (Supplementary Fig. S1).

It was recently reported that the R37G11-GAL4 driver labels cholinergic lateral horn neurons^15^. We confirmed these results and additionally examined whether the broader R48F03-GAL4 driver line also labels only cholinergic neurons. Indeed, ChAT-immunoreactivity exhibited an overlap with R37G11-GAL4 and R48F03-GAL4-labeled somata (Supplementary Fig. S1). Together with the complete lack of staining against dvGlut^36^ and GABA in PD2a1/b1 neurons of both lines, it is assumed that all cells described here are homogeneously cholinergic (Supplementary Fig. S1).

Previous reports have shown that PD2a1/b1 neurons receive input from a small and stereotypic number of PNs^15–17,21^. However, to what extent the population of all PD2a1/b1 neurons show a correlated response to this stereotyped input was not fully examined. To investigate this issue, we examined the population response of PD2a1/b1 neurons in the same animal, the correlation between PD2a1/b1 neuron odor responses, and the percentage of neurons activated by any given odor in the two GAL4 lines. Odor responses were measured in PD2a1/b1 somata rather than at PD2a1/b1 axons in order to be able to record simultaneously from a number of neurons. Twenty odors (R37G11-GAL4) and a subset of 15 odors (R48F03-GAL4) were selected and delivered at a 5 × 10^−2^ dilution (see Methods). Odors were chosen according to the Database of Odorant Responses (DoOR)^37^ to include odors predicted to activate PNs upstream to PD2a1/b1 neurons^15,17,21^ and, as controls, odors that are not predicted to activate the connected PNs.

**Figure 1:**
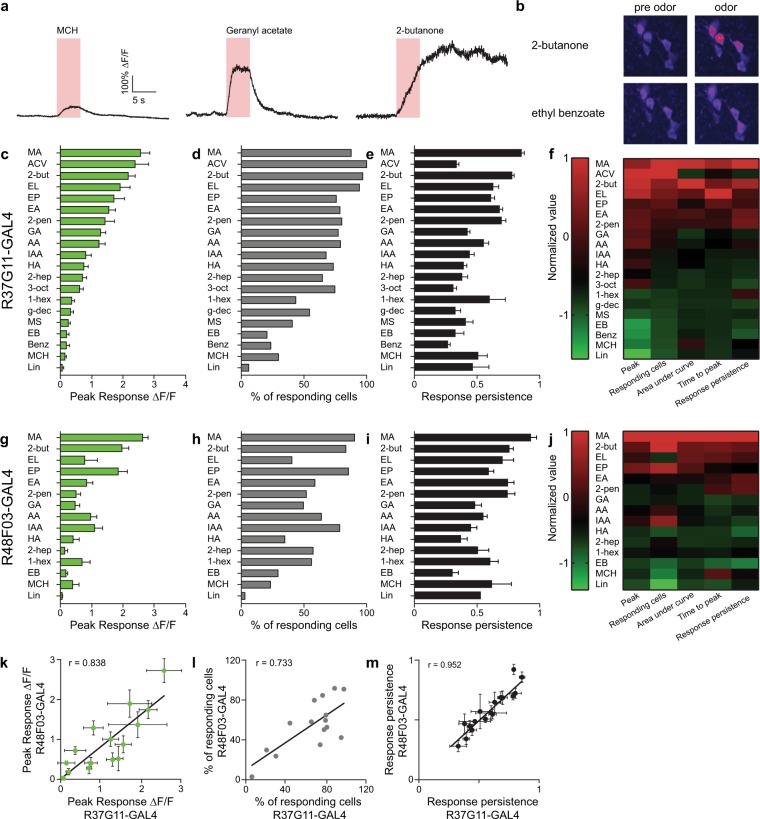
PD2a1/b1 neurons display variable odor responses to high odor concentrations. (**a**) Representative single odor response traces obtained from the same neuron. Different odor response dynamics and amplitudes were observed. Odor pulse is highlighted in red. (**b**) Representative activity maps demonstrating variable population odor responses in the same fly to 2-butanone and ethyl benzoate. Pre-odor pulse and during odor pulse activity maps are depicted. (**c,g**) Peak ∆F/F responses during odor response for GCaMP6f-labelled cells driven by drivers R37G11-GAL4 (**c**) and R48F03-GAL4 (**g**), respectively. (55 ≥ n ≥ 20 and 46 ≥ n ≥ 14 cells, respectively. At least 5 different flies were imaged from each genotype.) (**d,h**) Percentage of responding neurons obtained from all neurons from all flies pooled for cells labeled by R37G11-GAL4 (**d**) and R48F03-GAL4 (**h**). (55 ≥ n ≥ 20 and 46 ≥ n ≥ 14 cells, respectively. At least 5 different flies were imaged from each genotype.) (**e,i**) Response persistence for R37G11-GAL4 (**e**) and R48F03-GAL4 (**i**). Response persistence was measured as the inverse of the ratio between the maximal peak of the odor response and the average response 5 seconds after the maximal peak time. (55 ≥ n ≥ 20 and 46 ≥ n ≥ 14 cells, respectively. At least 5 different flies from each genotype were imaged.) (**f,j**) Heatmaps of normalized selected parameters: peak response, number of responding cells, area under the curve, time to peak, and response persistence for R37G11-GAL4 and R48F03-GAL4, respectively. To compare different odors across several parameters, we normalized the results by calculating a Z-score for each odor value. The calculated Z-scores were further divided by the maximal Z-score for each parameter to yield a maximum value of 1. In general, a high correlation was observed between the different parameters. (**k–m**) Correlation between the parameters obtained for R37G11-GAL4 and R48F03-GAL4 for peak ∆F/F (**k**), the percentage of responding neurons (**l**) and the response persistence (**m**). (See Supplementary Table S1 for statistical analysis).

PD2a1/b1 neurons exhibited varied response dynamics in their cell bodies. Some odors (e.g., geranyl acetate; Fig. 1a), elicited relatively rapid response kinetics, reaching peak response during the 5 second odor pulse. In contrast, other odors (e.g., 2-butanone; Fig. 1a), elicited relatively slow response kinetics, reaching peak response approximately 15 seconds after odor pulse onset, long after the end of the 5 second odor pulse. In addition, these prolonged odor responses often lasted throughout the subsequent 35 second recording. A similar variability was observed for neuron recruitment to an odor pulse, where certain odors activated almost all PD2a1/b1 neurons (e.g., 2-butanone, Fig. 1b), whereas others (e.g., ethyl benzoate, Fig. 1b) triggered a response in only a single neuron. Therefore, to better characterize the neuronal responses we analyzed five parameters: peak responses, percent of responding neurons, response persistence, time to peak, and the area under the curve (Fig. 1 and Supplementary Fig. S2, see methods).

Peak odor responses varied greatly between different odors. While some odors did not elicit any, or only a very mild, response, others produced very robust responses (Fig. 1c,g and Supplementary Fig. S2a,c). We found marked differences between the ranked order of odor responses elicited by a dilution of 2.5×10^−4^ as was previously described^15^ and those obtained by us for 5×10^−2^ (Fig. 1c). For example, whereas a 2.5×10^−4^ dilution of benzaldehyde, 4-methylcyclohexanol (MCH), and linalool evoked a relatively robust odor response compared to other odors examined^15^, a 5 × 10^−2^ dilution of these odors hardly activated any PD2a1/b1 neurons. Similar results were obtained for both the R37G11-GAL4 and R48F03-GAL4 driver lines, with a high correlation between the response amplitude in neurons labeled by the two lines (Fig. 1g,k and Supplementary Fig. S2).

To examine the percentage of neurons activated by each odor, we pooled all the neurons that responded to any of the odors (Fig. 1d). As with the peak amplitude, the responses spanned the entire range, from odors that triggered only a handful of neurons (i.e., 6% in the case of linalool), to those that activated all neurons (i.e., 100% in the case of ACV). Amplitude of odor responses, and the percentage of neurons responding, was tightly correlated (Fig. 1f,j and Supplementary Fig. S2g,h). Again, a high correlation was observed between the percentage of neurons responding in the two driver lines (Fig. 1h,j,l).

Although the response dynamics to different odors varied enormously, it was noticeable that odors with a longer time to peak and a high response persistence (Fig. 1a,e,f and Supplementary Fig. S2) also generated the strongest responses with respect to both amplitude and the number of activated neurons (Fig. 1e,f and Supplementary Fig. S2). However, it is important to note that strong responses were also observed to certain odors, such as geranyl acetate and ACV that had a relatively short time to peak and low response persistence. These response dynamics were also highly correlated between the two driver lines (Fig. 1i,m and Supplementary Fig. S2f). Taken together, PD2a1/b1 neurons exhibit varied responses to different odors. This heterogeneity was seen across all the criteria used for analysis with a high correlation found between the different criteria for a given odor (Fig. 1f,j and Supplementary Fig. S2).

### PD2a1/b1 neurons respond in a coordinated fashion

**Figure 2:**
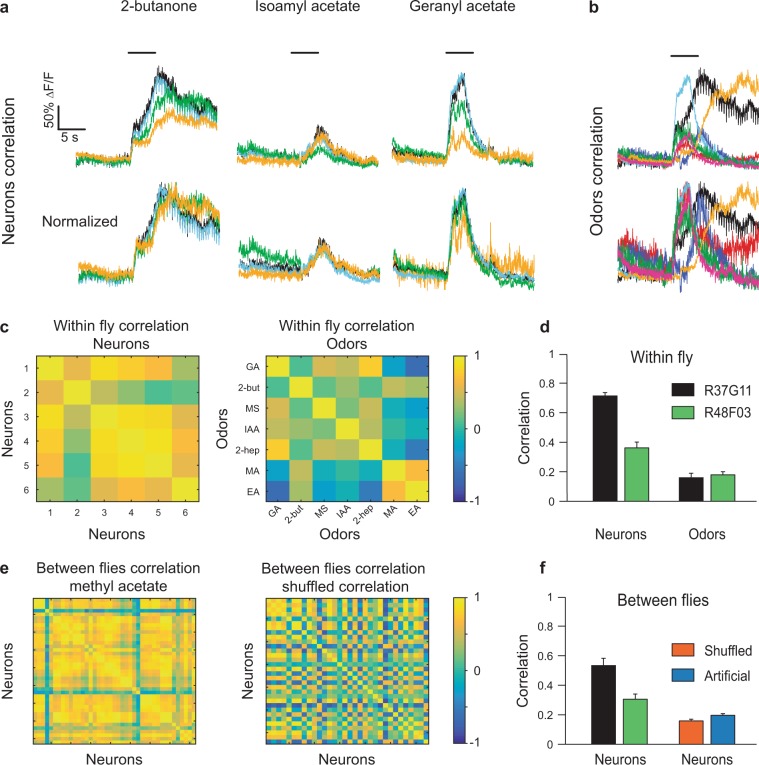
PD2a1/b1 neurons exhibit correlated population activity. **(a**) Odor responses from four PD2a1/b1 cell bodies in the same fly to three odors as indicated. A high correlation between the temporal pattern of the responses was observed. (**b**) Odor responses from one PD2a1/b1 neuron to seven odors. The temporal dynamics of the responses are not correlated. (**c**) *Left*, an example of the correlation values of six PD2a1/b1 neurons in the same fly obtained for the seven odors on the right. *Right*, an example of the correlation values of seven odors obtained for the six neurons on the left. (**d**) Within-fly mean correlation values for both driver lines (R37G11-GAL4 and R48F03-GAL4) for neurons and odors. High correlation values were obtained for the neurons and low correlation values for the odors (n = 26 flies and n = 9 flies, respectively). (**e**) *Left*, an example of the correlation values for a single odor obtained for all PD2a1/b1 neurons from all flies. *Right*, an example of the correlation values when the neuronal responses were shuffled (see Methods). (**f**) *Left*, between-flies mean correlation values for both driver lines (R37G11-GAL4, and R48F03-GAL4, n = 26 flies; the number of neurons ranged from 20 to 55 and n = 9 flies; the number of neurons ranged from 14 to 46, respectively). *Right*, Between-flies correlation for shuffled neurons and for artificial neurons (see Methods).

In order to investigate the population response of PD2a1/b1 neurons further, we first examined whether the PD2a1/b1 neurons in an individual fly respond in a coordinated fashion. To this end, we measured the correlation between the odor response vectors of each neuron to a given odor. When PD2a1/b1 neurons were covered by the R37G11-GAL4 driver line, there was a very high correlation between the neurons for a given odor (Fig. 2a,c). It should be noted that this was due to similar response dynamics, even though the amplitude of the response was very variable between the different neurons (Fig. 2a). Repeating this analysis for all odors and all flies, revealed an average correlation of 0.71 (Fig. 2d). We consider it possible that this high correlation between neurons was a consequence of the relatively low temporal accuracy of our recording (i.e., two-photon functional imaging) and that this method is not sensitive enough to capture subtle differences in response dynamics. We therefore also examined the correlation between different odor responses in the same neuron. As expected from the results presented in Fig. 1, there was no correlation in odor responses whatsoever (0.17 Fig. 2b–d). The stereotyped connectivity^15–17,21^ predicts that odor responses should also be correlated between flies and indeed, for a given odor, there was a high correlation (an average of 0.52, Fig. 2e,f) between the odor responses of all neurons of all flies (Fig. 2e,f). We took two approaches to ensure that this high correlation was not a consequence of our calculation method (see Methods). As a first measure, we reevaluated the correlation between the flies’ odor responses after shuffling the vectors. Since the correlation between different odors was ~0.2, it was expected that this procedure would yield the same result and indeed, the shuffled correlation was ~0.2 (Fig. 2e,f). As a second measure, in order to avoid the possibility that we generated a false high correlation by our selection of groups of correlated vectors, we generated an artificial data set with the same number of neurons, flies, and correlations between neurons of the same fly, but with no correlation between flies (the low odor correlation was used as the baseline for correlation). Again, this approach validated our calculations as only the low odor correlation was generated (Fig. 2f). Taken together, our results indicate that the small group of PD2a1/b1 neurons covered by the R37G11-GAL4 driver line exhibit correlated and stereotyped odor responses. Similar results were obtained for the PD2a1/b1 neurons covered by the R48F03-GAL4 driver line, although both the within-fly odor response correlations and consequently, the relationships in between-fly stereotyped odor responses, were much weaker.

Recently, different compartments in *Drosophila* neurons were discovered to exhibit different odor responses^38^. For example, the odor responses of Kenyon cell (KC) somata exhibit longer odor responses than KC dendrites, with the suggestion that these prolonged responses may hold the memory of a recently sensed odor^38^. To ensure that our analysis of PD2a1/b1 somata captures the essence of PD2a1/b1 neuronal output, we compared the odor responses obtained at PD2a1/b1 somata and axons. For this purpose, we selected an odor subset spanning the whole range of observed odor peak responses and response kinetics (Fig. 1). As expected from the pseudo-unipolar morphology of invertebrate interneurons, odor responses in PD2a1/b1 somata were low-pass filtered (Supplementary Fig. S3a). Nevertheless, we also seemed to capture relatively rapid response dynamics, albeit at a lower magnitude. For example, 2-butanone elicited a biphasic response in axons, with a small and fast response on odor onset, followed by a much stronger and slower response following odor offset. This biphasic response, although heavily filtered, was still observed in PD2a1/b1 somata (Supplementary Fig. S3a). As expected, different odors elicited statistically different responses for all three parameters examined. However, for a given odor, the responses in the axonal compartment were only slightly stronger than those in the somata (except for ACV, Supplementary Fig. S3b,c). In contrast, the response dynamics were markedly shorter in the axons than in the PD2a1/b1 somata (Supplementary Fig. S3d–g). In conclusion, the responses recorded in the somata tended to be slower, but still followed the same kinetics observed in the axon compartment.

### PD2a1/b1 neurons contribute to aversive responses to odors

**Figure 3:**
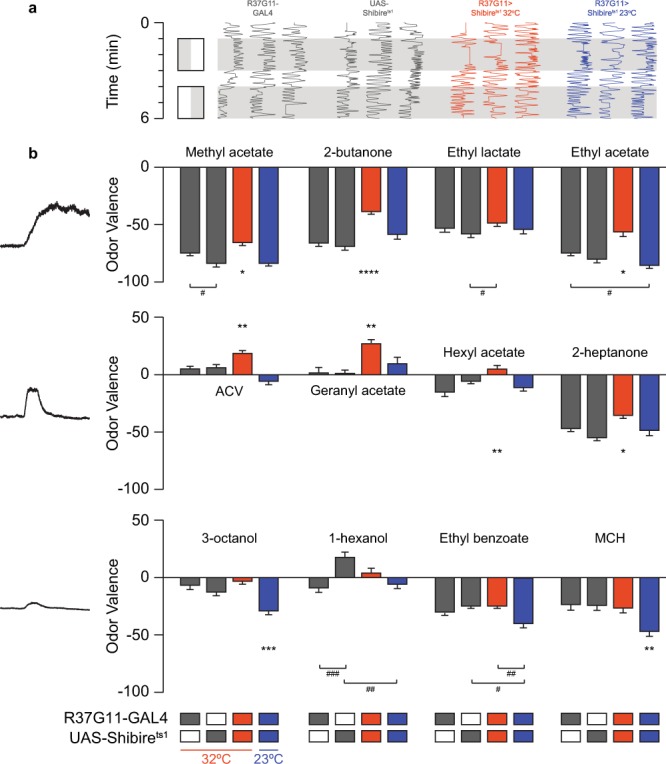
PD2a1/b1 neurons contribute to aversive responses to high odor concentrations. (**a**) An example of traces obtained for naïve flies for 2-butanone. Gray, parental control groups; red, the experimental group at 32 °C; blue, the experimental control group at 23 °C. Naïve response to an odor (gray) was tested against mineral oil (white). (**b**) Mean valence scores (see Methods) from experiments as in **a**, for the designated odors. Odors are arranged according to their response profile as in Fig. 2 and Supplementary Fig. S2. A consistent shift towards more positive valence values was observed during PD2a1/b1 inhibition for all odors that activate PD2a1/b1 neurons, except for ethyl lactate. No effect during PD2a1/b1 inhibition was observed for odors that only weakly activate PD2a1/b1 neurons. (137 ≥ n ≥ 21 flies for all conditions, * indicates a significant difference of the indicated group from all other groups according to a multiple comparison test. The lowest value of all multiple comparison tests is presented. # indicates a significant difference only between the two indicated groups. *, ^#^p < 0.05, **, ^##^p < 0.01, ^###^p < 0.0005, ****p < 0.0001, see Supplementary Table S1 for statistical analysis).

Based on our results, we selected 12 odors that matched three odor categories: odors that elicited strong and prolonged neuronal responses (methyl acetate, 2-butanone, ethyl lactate, ethyl acetate), odors that elicited strong but short neuronal responses (ACV, geranyl acetate, hexyl acetate, 2-heptanone), or odors that generated only weak neuronal responses (3-octanol, 1-hexanol, ethyl benzoate, MCH) in PD2a1/b1 neurons. We then examined whether silencing PD2a1/b1 neuronal output by expressing UAS-Shibire^ts1^ using the R37G11-GAL4 driver line, affected the valence attached to these odors at 10^−2^ odor dilution in satiated flies. For the behavioral experiments, flies are placed in linear chambers where they can walk back and forth only along one axis and odors are delivered from both sides of the chamber with a clear decision area in the middle of the chamber (Fig. 3a). The flies in this apparatus constantly walk around the chambers and thus are making repeated decisions (see methods). All odors examined at a 10^−2^ dilution, elicited either avoidance or neutral behavioral responses. For 7 out of the 12 odors examined: methyl acetate, 2-butanone, ethyl acetate, ACV, geranyl acetate, hexyl acetate, and 2-heptanone, which elicited strong peak responses (Fig. 1), silencing PD2a1/b1 neurons caused a significant decrease in odor avoidance behavior (Fig. 3, Supplementary Table S1, which contains detailed statistical comparisons and tests, including all controls, for all figures). Interestingly, despite eliciting strong neuronal responses in PD2a1/b1 neurons, one odor (ethyl lactate, Fig. 3) exhibited no change in odor valence. For the odors that elicit weak peak responses (3-octanol, 1-hexanol, ethyl benzoate, and MCH, Fig. 1), we did not observe any significant change in odor valence (Fig. 3). However, it is important to note that these odors became less aversive, owing to the temperature increase, which might mask possible effects on valence. Although the R37G11-GAL4 driver line had almost no label in the thoracico-abdominal ganglia, we still verified that fly movement was not impaired in R37G11-GAL4 flies expressing UAS-Shibire^ts1^. Walking speed analysis revealed no defects in the flies’ locomotion (Supplementary Fig. S4a). In addition, we obtained similar behavioral results with a sparser split-GAL4 line, LH989^15,18^ (see methods) which is based on the R37G11-ZpGdbd and R29G05-p65ADZp promoter fragments (Supplementary Fig. S4b). These results suggest that PD2a1/b1 neurons mediate avoidance behavior to odors in the case of satiated flies at the relatively high odor concentration of 10^−2^ used in this experiment.

### Odor concentration modulates the effect of PD2a1/b1 neurons on behavioral output

**Figure 4:**
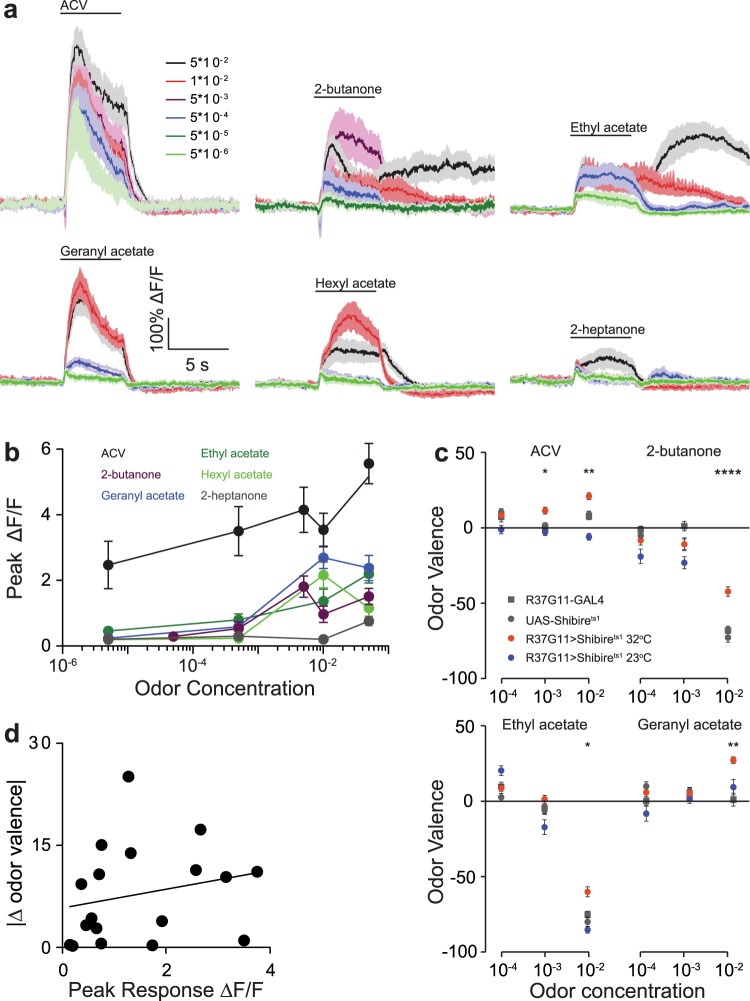
PD2a1/b1 neurons do not contribute to aversive responses at low odor concentrations. (**a**) Average traces obtained from PD2a1/b1 axonal projections in response to increased concentrations of the designated odors. For 2-butanone and ethyl acetate, a change in the temporal dynamics of the odor response was observed at high odor concentrations. (17 ≥ n ≥ 6 flies for all odors). (**b**) Peak response analysis of the data presented in **a**. Except for ACV and 2-heptanone, saturation in odor response was observed at 1×10^−2^ odor concentration (see Supplementary Table S1 for statistical analysis). (**c**) Mean valence scores (see Methods), for the designated odors (results for an odor concentration of 1×10^−2^ as obtained from Fig. 2 and are presented for comparison). In general (except for 10^−3^ ACV) no effect of PD2a1/b1 neurons was observed at low odor concentrations (137 ≥ n ≥ 21 flies for all conditions, *p < 0.05, **p < 0.01, ****p < 0.0001, see Supplementary Table S1 for statistical analysis). (**d**) Absolute value of the magnitude of the valence change as a function of the mean peak response. Valence change was calculated as the smallest difference from the control groups (parental and temperature). No significant correlation was found (R = 0.228, p-value = 0.3617).

This avoidance behavior raises the question of whether the PD2a1/b1 effect on valence is concentration dependent. Since PD2a1/b1 neuronal odor responses increase linearly with odor concentration^21^, we first verified that lower odor concentrations do elicit a response. Two-photon functional imaging was used to examine the neuronal responses of PD2a1/b1 neurons to varying concentrations of six odors for which silencing PD2a1/b1 neurons resulted in a phenotypic change. As previously described^21^, the results indicated a concentration-dependent increase in the odor response of PD2a1/b1 neurons to all odors examined (Fig. 4a,b). In most cases we observed saturation of the odor response (with no difference between odor responses at 1 × 10^−2^ and 5 × 10^−2^). Two exceptions were 2-heptanone and apple cider vinegar (ACV) where there remained a significant increase in odor response with increasing odor concentration (Fig. 4b) even at the highest concentrations. In addition to the quantitative changes in neuronal response to different odor concentrations, certain odors (2-butanone and ethyl acetate, Fig. 4a) exhibited a dramatic change in the temporal dynamics. Based on this characterization, we selected four odors whose valence was affected by silencing PD2a1/b1 neurons (Fig. 3), and which produced neuronal odor responses at lower concentrations (Fig. 4a,b). Silencing PD2a1/b1 neurons had no effect on odor avoidance behavior at any of the odor concentrations tested, except for 10^−3^ ACV, which resulted in a significant decrease in avoidance behavior (Fig. 4c). This apparent lack of contribution by PD2a1/b1 neurons to odor valence could not be attributed to a lack of ability to detect the odors, as the flies could be conditioned against the low odor concentration (Supplementary Fig. S5). The disappearance of the PD2a1/b1 effect on odor valence at lower odor concentration was surprising because of the high neural activity observed in PD2a1/b1 neurons even at low concentrations (ACV, Fig. 4a,b). Indeed, pooling the behavioral results presented in Figs. 3 and 4c, revealed no correlation between the magnitude of odor response observed in PD2a1/b1 neurons and the extent of the effect seen on behavior (Fig. 4d). In conclusion, the contribution of PD2a1/b1 neurons to odor avoidance behavior is concentration dependent.

### Starvation modulates the effect of PD2a1/b1 neurons on behavioral output but not their odor response

**Figure 5:**
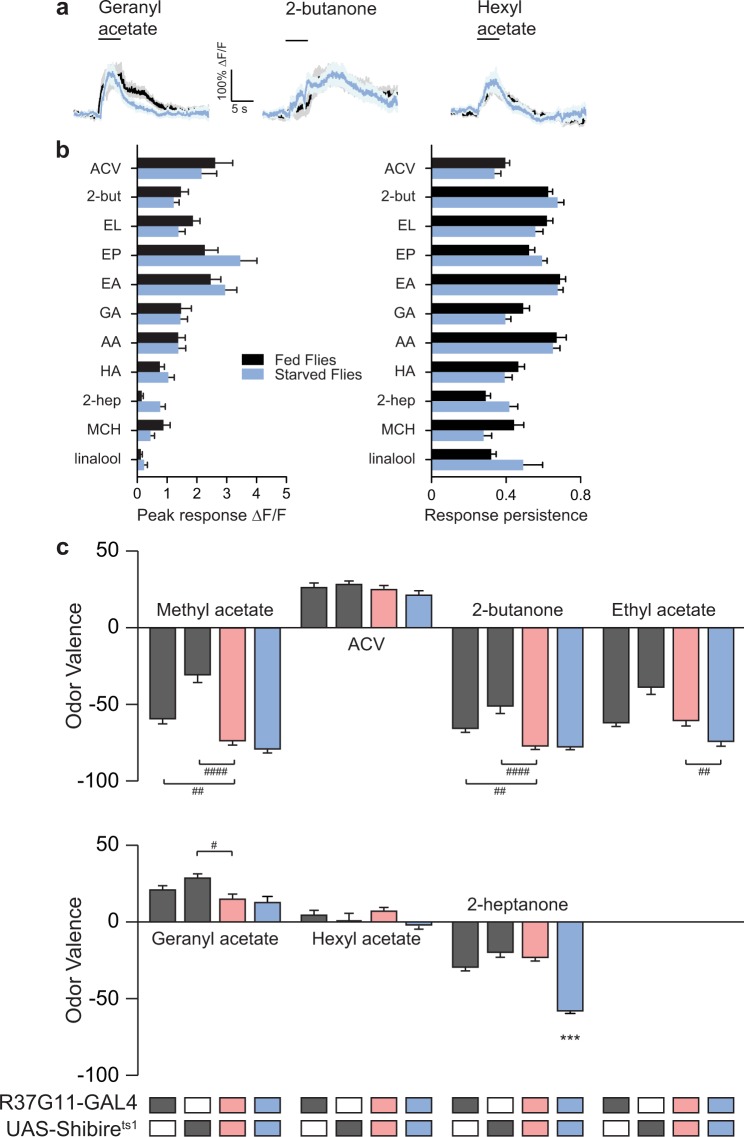
Starvation abolishes the effect of PD2a1/b1 neurons on behavioral output without changing their odor responses. (**a**) Averaged traces of odor responses for fed (black) and starved (light blue) flies; no effect of starvation was observed. (**b**) Analysis of peak odor responses and response persistence for the designated odors in fed and starved flies. No statistical difference was observed (49 ≥ n ≥ 18 cells, from at least 5 different flies; see Supplementary Table S1 for statistical analysis). (**c**) Mean valence scores of starved flies for the designated odor. Gray, parental controls; light red, the experimental group at 32 °C; blue, the experimental control group at 23 °C. Starvation abolished the effect of silencing PD2a1/b1 neurons. (87 ≥ n ≥ 47 flies, *indicates a significant difference of the indicated group from all other groups according to a multiple comparison test. The lowest value of all multiple comparison tests is presented. ^#^indicates a significant difference only between the two indicated groups. ^#^p < 0.05, ^##^p < 0.01, ^***^p < 0.0005, ^####^p < 0.0001, see Supplementary Table S1 for statistical analysis.).

In addition to odor concentration, there are a number of other factors that may affect the neural activity and the contribution to odor valence by PD2a1/b1 neurons. One such factor is the satiation state of the flies. Starvation has been shown to enhance attraction to food odors^31,39,40^ as well as modulate the neural activity in various glomeruli^31^. In particular, the activity in PNs innervating glomeruli DM1 and DM4 (which are presynaptic to PD2a1/b1 neurons) has been reported to increase following starvation^31,41^. In order to investigate the effect of context as manifested by starvation on the contribution of PD2a1/b1 neurons to odor valence we first examined whether starvation affects PD2a1/b1 neuron odor responses. With one exception (time to peak with acetic acid, Fig. 5a,b and Supplementary Fig. S6), there was no significant difference in any of the parameters analyzed. We then selected the seven odors that were affected by silencing PD2a1/b1 neurons in satiated flies (Fig. 3), and examined the effect of PD2a1/b1 neurons on behavioral responses of starved flies at 10^−2^ odor concentration. Surprisingly, although starvation did not affect PD2a1/b1 neuronal odor responses, it completely suppressed the previously observed behavioral effects in fed flies, so that the valence values for all tested odors remained unchanged after silencing PD2a1/b1 neurons (Fig. 5c) compared to controls. In the case of 2-heptanone we also observed a strong temperature effect in the starved flies. The reason for this observation remains unknown although it could be associated with the reports that heat reduces olfactory sensitivity^42^ or that temperature can generate increased odor attraction^39^. Taken together, our results indicate that context, as manifested by starvation, diminishes the contribution of PD2a1/b1 neurons to odor avoidance behavior but without modulating the neuronal odor responses in the somata.

### The effect of PD2a1/b1 neurons on odor valence develops with time

**Figure 6:**
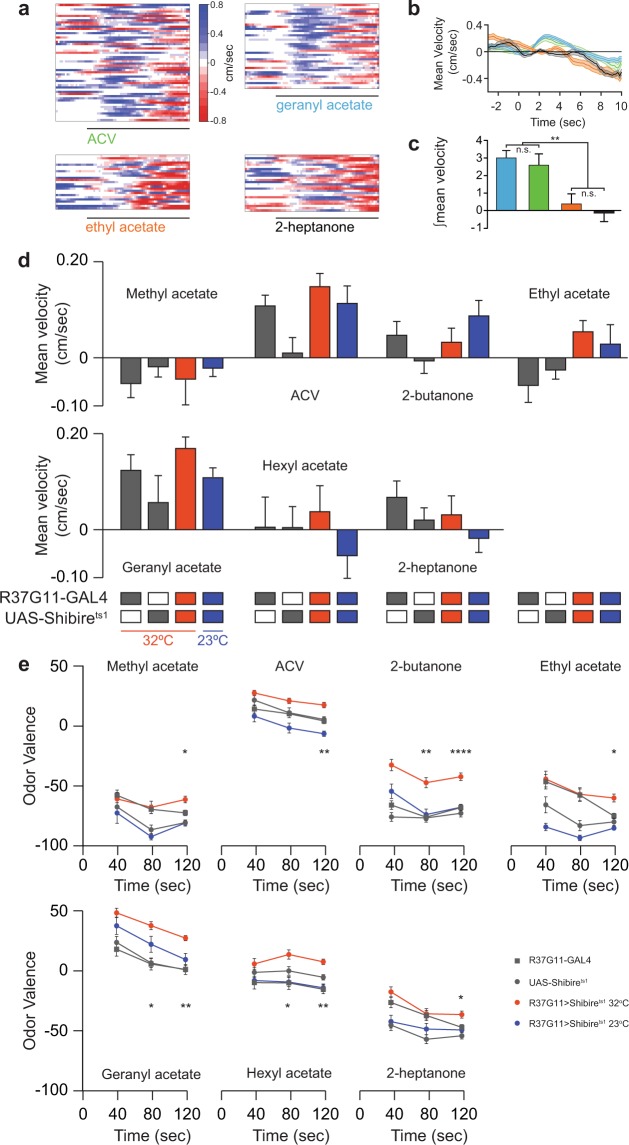
The effect of PD2a1/b1 neurons on odor valence develops with time. (**a**) Heat maps of the initial walking velocity of single flies at the onset of the odor pulse, 3 seconds prior to the odor pulse, and 10 seconds following the odor pulse (black line). A positive velocity indicates movement towards the odor pulse. For the attractive odors (ACV and geranyl acetate), a clear positive velocity was observed for most flies. (**b**) Mean velocity of the heat maps presented in **a**. A clear movement towards the odor source was observed for the attractive odors. Odors are according to the color coding in panel **a**. (**c**) Analysis of the integral of the response for the flies presented in **a** for the first 3 seconds following odor onset. Odors are according to the color coding in panel **a** (** p < 0.01, see Supplementary Table S1 for statistical analysis). (**d**) Mean velocity scores during the first 3 seconds following odor onset for the designated odor. Gray, parental controls; red, the experimental group at 32 °C; blue, the experimental control group at 23 °C. No significant difference was observed (53 ≥ n ≥ 18 flies). (**e**) Mean valence scores (see Methods) obtained over time for the designated odors. Gray, parental controls; red, the experimental group at 32 °C; blue, the experimental control group at 23 °C. PD2a1/b1 neurons contribution to odor valence develops with time. Values at 120 seconds were similar to those presented in Fig. 2 (155 ≥ n ≥ 17 flies for all conditions, *p < 0.05, **p < 0.01, ****p < 0.0001, see Supplementary Table S1 for statistical analysis.).

The results obtained for starved flies and different odor concentrations indicate that the contribution of PD2a1/b1 neurons to odor avoidance behavior is varied and inconsistent. Another parameter that may affect the contribution of these neurons to odor avoidance is the temporal dynamics of the odor response. Since odor responses of ORNs and PNs are known to change over time^43–46^, it was of interest to examine PD2a1/b1 neurons effect on valence with time. As our behavioral apparatus provides constant tracking of the flies, we were able to re-analyze the data at different time points. We first analyzed the velocity at the first exposure to the odor (positive values represent movement towards the odor source, see methods), and verified that this analysis indeed captures flies’ preferences as previously reported^47,48^. For attractive odors, such as ACV and geranyl acetate, we observed an initial movement towards the odor source. The velocity of this movement was significantly reduced towards 2-heptanone and ethyl acetate, which generate aversion in our behavioral assay (Fig. 6a–c). When examined across all odors and genotypes, silencing PD2a1/b1 neurons had no effect on the initial preference to any odor that generated an effect after 120 seconds exposure (compare Figs. 3 and 6d). These results suggest that while PD2a1/b1 neurons do not affect the initial preference for an odor, an effect might develop over time. When we re-analyzed our data for the seven odors selected previously, at two intermediate time points (Fig. 6e), the results appeared to support this hypothesis. While 40 seconds exposure produced no significant effect on odor valence, after 80 seconds exposure, three of the seven odors examined did display a significant effect (Fig. 6e). Thus, the contribution of PD2a1/b1 neurons to odor valence is also dependent on the time of odor exposure. Odor conditioning has already been used to demonstrate the development of an effect of PD2a1/b1 neurons on odor valence with time^15^, however, in that case, the time scale was measured in hours, as opposed to the current changes in naïve behavior, which occurred within seconds.

### PD2a1/b1 neuron output can interfere with aversive conditioning

We have found that PD2a1/b1 neurons contribute to aversion even though they also receive input from glomeruli reported to contribute to attraction. We therefore sought to verify our results using another assay. To this end, we used classical olfactory conditioning^49–54^ (Supplementary Fig. S7a). In this assay, an odor is assigned a negative value by pairing it with an electric shock (conditioned odor, CS^+^) and is compared to another odor (unconditioned odor, CS^−^). Electric shock pairing adds a learned behavioral drive to the original naïve odor valence. If there is a strong naïve aversion to the CS^−^, it is possible that the learned aversion towards the CS^+^ will not be apparent above the naïve aversion. We reasoned that in such a case silencing PD2a1/b1 neurons might improve aversive conditioning by decreasing the naïve aversion of the CS^−^. Out of the odors we tested, only ethyl lactate elicited a profound odor response in PD2a1/b1 neurons (Fig. 1) without a change in valence after silencing (Fig. 3). We therefore selected ethyl lactate as the CS^+^ and 2-butanone as the CS^−^ whose avoidance is decreased when PD2a1/b1 neurons are silenced (Fig. 3). Prior to conditioning flies robustly avoided the CS^−^, 2-butanone (Supplementary Fig. S7b) and, following conditioning, control flies still avoided the CS^−^ (Supplementary Fig. S7b). Thus, the conflict between learned and naïve drives resulted in only a weak change in avoidance (Supplementary Fig. S7b). However, silencing PD2a1/b1 neurons, which reduces the CS^−^ naïve aversion (Fig. 3), dramatically increased the change in avoidance, allowing them to better avoid the CS^+^ (Supplementary Fig. S7b). This enhanced conditioning capability was not due to a change in odor discrimination, since naïve flies showed no change in odor preference between 2-butanone and ethyl lactate even when PD2a1/b1 neurons were silenced (Supplementary Fig. S7b, pre-training avoidance).

As a control, we used ethyl lactate as the CS^−^ since it does not change its valence value following the silencing of PD2a1/b1 neurons (Fig. 3), together with geranyl acetate as the CS^+^. As expected, prior to conditioning flies robustly avoided the CS^−^, ethyl lactate (Supplementary Fig. S7b) and after conditioning, control flies still avoided the CS^−^ although to a slightly lesser extent than seen in the previous odor pair (Supplementary Fig. S7b). However, in contrast to the observations made with the 2-butanone/ethyl lactate odor pair, silencing of PD2a1/b1 neurons did not cause any change in avoidance for the ethyl lactate/geranyl acetate odor pair (Supplementary Fig. S7b). This is consistent with the observation that ethyl lactate exhibited no change in odor valence when PD2a1/b1 neurons were silenced. Taken together, we can conclude that silencing PD2a1/b1 neurons can contribute to aversive conditioning. This contribution is probably not a result of the direct participation of these neurons in the conditioning process, as was recently demonstrated^15^, but is more likely to be due to changes in the innate odor valence of the chemical compounds.

### PD2a1/b1 downstream neurons include MB KCs and neurons in the fan shaped body

Our results suggest that context as manifested by a range of parameters (i.e. satiation, concentration, and odor exposure time) affects the contribution of PD2a1/b1 neurons to odor valence. This probably occurs without affecting the neural activity of PD2a1/b1 neurons themselves (satiation), or with no correlation between neural activity and the extent of their effect (concentration). These observations thus suggest that context is integrated downstream of the PD2a1/b1 neurons.

**Figure 7:**
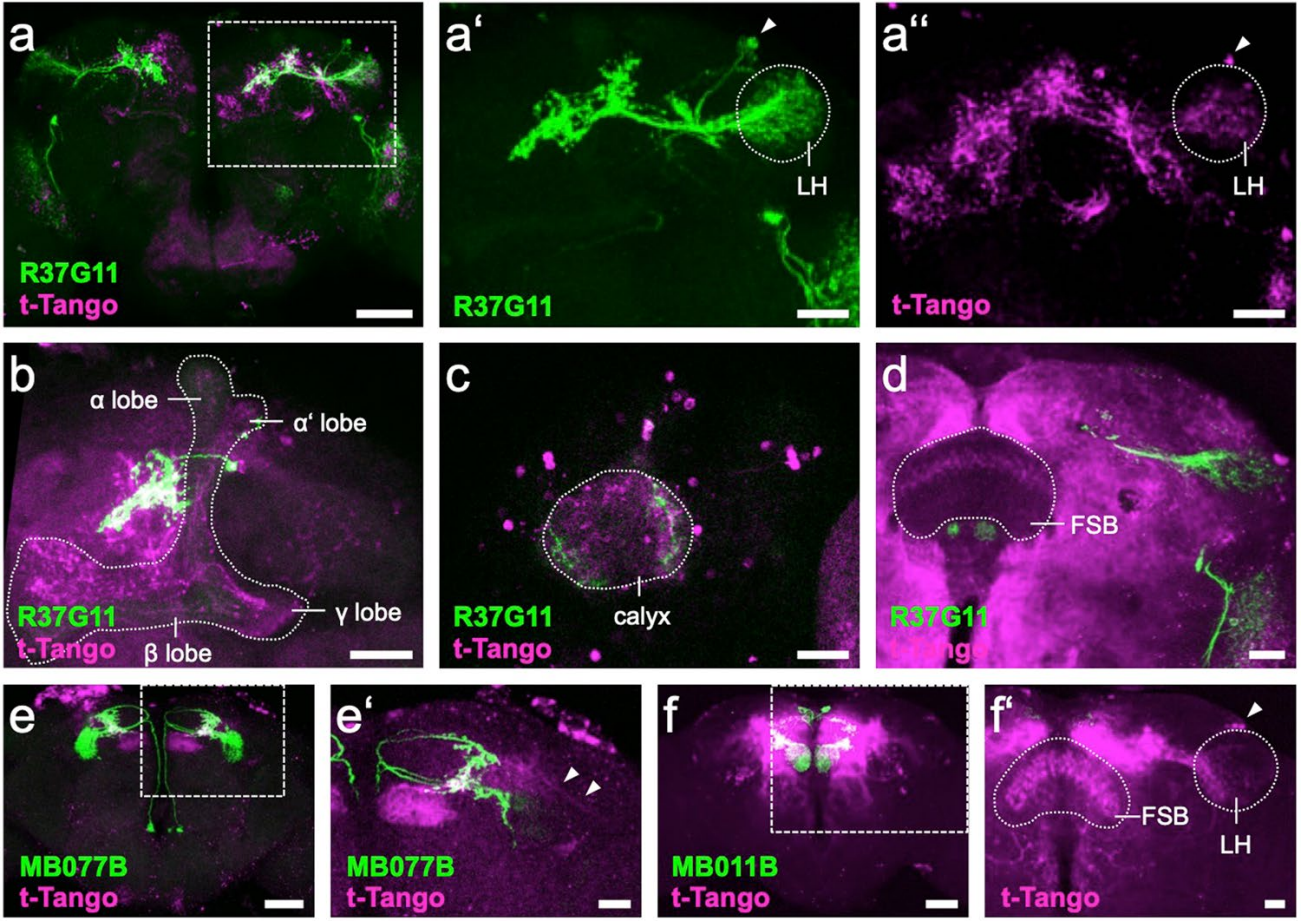
*trans*-Tango downstream targets of PD2a1/b1, MBON- 𝛾2α’1 and MBON- β’2mp neurons. (**a**) R37G11-GAL4-driven myrGFP (green) labels 6-7 cells in the LH, whereas the *trans*-Tango signal (magenta) appears to be strongest in overlapping neuropil regions and in random sparse KC subsets in the MB (boxed area enlarged in a’ and a”). (**a’**) R37G11-GAL4-driven myrGFP (green) labels 6-7 cells (arrowhead) in the LH (stippled). (**a”**) R37G11-GAL4-driven *trans*-Tango signal (magenta) marks cells (arrowhead) in the LH (stippled). (**b**) Confocal stack of MB (stippled), highlighting the PD2a1/b1 neurites in proximity to the vertical lobe and a *trans*-Tango signal in all sublobes, i.e., αβ, αβ’, and 𝛾. (**c**) Confocal stack of MB calyx (stippled) showing innervation by R37G11-GAL4-driven GFP (green) and *trans*-Tango signal in both KC somata and the calyx neuropil. (**d**) Confocal stack of left hemibrain highlighting R37G11-GAL4-driven myrGFP (green) and postsynaptic *trans*-Tango signal in layer 6 of the FSB (stippled). (**e**) MB077B-GAL4-driven myrGFP (green) labels 3-4 MBON-𝛾2α’1 neurons, whereas a fairly restricted putative downstream *trans*-Tango signal (magenta) most prominently labels MBON- β’2mp (boxed area enlarged in e’). (**e’**) Confocal stack of left hemibrain highlighting MB077B-GAL4-driven mCD8::GFP (green) and postsynaptic *trans*-Tango signal (magenta) in β’2mp of the MB and in putative LHON neurites (arrowheads). (**f**) MB011B-GAL4-driven myrGFP (green) labels MBON- 𝛾5β2α and MBON- β’2mp, whereas the putative downstream *trans*-Tango signal (magenta) labels several different cell types (boxed area enlarged in f’). (**f’**) Confocal stack of right hemisphere highlighting the postsynaptic signal of MBON- β’2mp in PD2 LHONs (arrowhead) in the LH (stippled) and in layer 4 & 5 of the FSB (stippled). (**a–f’**): Confocal stack views of 1-2 µm-thick optical section data. Scale bars: **a,e,f**: 50 µm; **a’,a”,b-d,e’,f’**: 20 µm.

Recent EM data did not clearly locate potential downstream neurons^15,18^, although PD2a1/b1 axons were thought to be in close proximity to the axons of three MBONs (γ2α‘1, α‘2, and β‘2 mp), which may suggest a common downstream integrator. Hence we attempted to identify downstream targets of PD2a1/b1 using *trans*-Tango^55^ with R37G11-GAL4. This labeled mostly other LH neurons including PD2a1/b1 neurons (Fig. 7a-a”), in accordance with the report that 18.4% of the input synapses are reciprocal between PD2a1/b1 LH output neurons^15^. Additionally, strong *trans*-Tango signals appeared in varying subsets of all three main Kenyon cell types (Fig. 7b,c). The only site that allows for direct interaction of γ KCs with R37G11-GAL4-expressing neurons is the MB calyx, which is innervated by PD2b1 neurons^15^. We therefore examined the cellular polarity of these contacts. R37G11-GAL4 expression of the dendritic marker DenMark^56^, and the synaptic marker Dsyd-1^57^ indeed suggests the presence of presynaptic sites of PD2b1 in the MB calyx (Supplementary Fig. S8). Furthermore, PD2b1 neurites exhibited colocalization with ChAT-immunoreactivity in the calyx (Supplementary Fig. S9), supporting the argument for active synapses and in line with the cholinergic identity of this cell type (Supplementary Fig. S1). Despite all these indications, we are very cautious about deducing functional calycal connectivity between PD2b1 neurons and KCs, given that a previous EM study^15^ did not report any presynapses between PD2b1 neurons and KCs in the calyx of their examined specimen.

Functionally, a good candidate site for downstream integration of olfactory information independently processed by the MB and LH would be a premotor center like the fan-shaped body (FSB). Indeed, in some preparations we did see a *trans*-Tango signal driven by R37G11-GAL4 in layer 6 of the FSB (Fig. 7d). We should note, however, that the *trans*-Tango signal in the FSB was less prominent than the label in reciprocal LH cells and MB KCs. Recent data suggest that the FSB layer 6 is downstream of at least two MBON types (MBON-α’2 and MBON-β2β’2α)^58^, with yet another MBON apparently synapsing onto the FSB in layers 4 & 5 (MBON-β’2mp)^58^.

To further investigate the FSB as a potential downstream integrator region, we performed *trans*-Tango experiments with MBON-γ2α‘1 as these neurons arborize in close proximity with those of PD2a1/b1 neurons^15^. Surprisingly, instead of FSB labeling, we identified MBON-β‘2mp as a postsynaptic target of MBON-γ2α‘1 (Fig. 7e’). We also observed weak labeling in putative LHONs, which is in accordance with the recent report of axoaxonic contacts of MBON-γ2α‘1 onto the LHON AD1b2^18^. These results suggest that not only MBON-α‘2, but also MBON-γ2α‘1 converges onto MBON-β‘2mp, making it an integrator of highly processed MB output. A repetition of the *trans*-Tango experiment with MBON-β‘2mp^58^ without antibody staining confirmed FSB labeling (Fig. 7f,f’). Additional *trans*-Tango labeling was observed in PD2 LHONs, but in contrast to the case with PD2a1/b1 neurons, the signal was mainly associated with arborizations in the ventral LH (Fig. 7f’). Taken together, our results suggest that LH neurons, KCs, and FSB neurons are possible downstream integrators of PD2a1/b1 neurons and that MB-processed information is returned to the LH via MBON-β‘2mp.

## Discussion

The results described here indicate that PD2a1/b1 neurons can mediate aversive behavioral responses of satiated flies to odor at the relatively high concentration of 10^−2^. This effect is context dependent and is influenced by odor identity (even between odors with a similar activation pattern), odor concentration, exposure time, and fly satiation. Interestingly, starvation blocks the PD2a1/b1 aversive effect but not by directly affecting the activity of PD2a1/b1 neurons.

The temporal dynamics of the PD2a1/b1 neuron response vary dramatically, with some odors eliciting a prolonged and robust response but only at high odor concentrations. The most likely explanation for this prolonged odor response is that this is not an intrinsic property of PD2a1/b1 neurons but can rather be attributed to properties of the ORNs^59^. The most likely candidate is OR59b, which, according to the DoOR database, is the OR shared by all odors that generate a prolonged odor response^37^. This notion is also supported by results indicating prolonged odor responses to ethyl acetate in DM4, the glomerulus cognate to OR59b^46^, which was shown to be connected to PD2a1/b1 cells^15,17^.

The results presented here concerning the role of PD2a1/b1 neurons in driving aversive behavioral responses to odors, seem to contradict a previous report that claimed that PD2a1/b1 neurons exclusively drive attraction^15^. It is important to note that this previous study used 3 day old flies for the behavioral experiments, rather than the 7–14 day old flies used here. However, this difference is unlikely to explain the difference in the effects on naïve odor valence observed in the two studies following silencing of PD2a1/b1 neurons. This apparent discrepancy may be explained if PD2a1/b1 neurons reinforce a valence value that is generated by other neurons in the fly’s brain. Thus, for an odor with a positive valence value, indicating attraction, PD2a1/b1 neurons further strengthen the attractive response. Conversely, for an odor with a negative value, indicating aversion, PD2a1/b1 neurons further strengthen the aversive response. If this is indeed the case, silencing PD2a1/b1 neuronal output should push the flies towards a random choice (i.e., 0 preference index) rather than driving either attraction or aversion. However, the results obtained with ACV and geranyl acetate negate this option. Satiated flies exposed to high concentrations of ACV or geranyl acetate showed no preference to the odor and had a preference index around zero (Fig. 3). However, silencing PD2a1/b1 neurons resulted in a marked increase in attraction towards these odors, pushing the preference index to positive values.

Another possible explanation for the apparent discrepancy in results may be the different behavioral approaches (with some marked differences) used in the two studies. Specifically, flies in behavioral chambers (as used here) are in constant motion, so that they repeatedly enter and exit the odor plume, and make multiple decisions each time^54,60,61^. In contrast, flies in the T-maze (as used in^15^) make fewer decisions and reach steady state more rapidly^62^. Another difference is that flies in a T-maze make their decision within 3–4 seconds^63^, whereas the standard analysis of the behavioral chambers takes into account a two minute window with repeated decisions^54,60,61^. Given the strong context dependent effect of PD2a1/b1 neurons it is very plausible that PD2a1/b1 neurons contribute to both attraction and aversion. Consistent with this notion, while we did not identify a role for PD2a1/b1 neurons in attraction, starvation and low odor concentration abolished the aversive effect of activating these neurons. In addition both studies demonstrated that PD2a1/b1 neurons contribute differentially to odor valence even if the neural activity elicited by the odors is similar (Figs. 1 and 3). In this context, it is important to note that optogenetic activation of PD2a1/b1 neurons resulted in a mild yet significant aversive response^18^. Thus, the combined results of our study and those of Dolan *et al*.^15,18^ suggest that PD2a1/b1 neurons may affect odor valence in a number of apparently contradictory ways. This conclusion is further supported by recent results demonstrating that PD2a1 neurons are essential for context-dependent long term memory, but not for “classical” MB-mediated, context-independent long term memory^64^.

PN input to PD2a1/b1 neurons consists of neurons innervating predominantly six glomeruli: DM1, DM4, VA2, VM3, DP1m, and DP1l^15,17^. These glomeruli are considered to drive attraction^15^ as they are activated by appetitive and food odors^65–67^. This raises the question of how an input, which presumably generates an attraction behavior, can generate an aversive response to odor. However, these glomeruli were also reported to drive aversion (VM3^66^), to respond to aversive odors (DM4, VA2^65^) or to have no effect on attraction (DM4^67^). Furthermore, it was suggested that in the case of *Drosophila* larvae, hyperactivation of Or42b neurons, which are the cognate neurons to glomerulus DM1 can trigger repulsion from odors^68^. Thus, it is possible that glomeruli that innervate PD2a1/b1 neurons also participate in aversive behavioral responses to odors.

How can a single neuron class generate opposing behavioral responses? Without knowledge and genetic access to the downstream neurons, this is difficult to answer. However, one plausible explanation is that recruitment of downstream neuron types depends heavily on PD2a1/b1 neuronal activity. Thus, a low odor concentration that activates PD2a1/b1 neurons only weakly, may efficiently recruit one neuronal pathway leading to attraction. In contrast, a high odor concentration that produces a robust activation of PD2a1/b1 neurons may recruit a different neuronal pathway leading to aversion. This notion was demonstrated with salt, where low and high concentrations of salt, (leading to attraction and aversion, respectively), are encoded by different neuronal pathways^69^. Similarly, while a low concentration of ACV triggers innate attraction by activating two glomeruli, DM1 and VA2, a higher concentration of ACV recruits an additional glomerulus, DM5, which leads to reduced attraction^67^. For mechanosensory processing, the bandpass filtering mechanism of *Drosophila* antennal vibration sensation requires downstream neurons to have different levels of voltage-gated channels and different membrane resting potentials^70^.

What may be the neurons downstream of PD2a1/b1 neurons? Unfortunately, the *trans*-Tango experiments did not provide a clear identification of the neurons downstream of PD2a1/b1. In accordance with a previous EM analysis^15^, we saw strong labeling of LH neurons, but in contrast, our experiment also detected a signal in the MB, although the labeled subsets were not consistent. We did not detect dopaminergic neurons or MBONs, which were suggested to be innervated by PD2a1/b1 neurons. In some preparations we detected *trans*-Tango labeling in layer 6 of the FSB. Together with the recently described *trans*-Tango label in the same layer by MBONs^58^, this would imply a downstream integrator of both MB and LH information in a premotor center. The FSB would represent an ideal candidate for a downstream integration site for olfactory information processed independently by both the MB and the LH. As part of the central complex, the FSB belongs to the main premotor center of the central brain, analogous to the basal ganglia in vertebrates^71^. The central complex itself serves as the main hub, integrating innate and conditioned multisensory information to relay behavioral decisions to the respective downstream effectors^71,72^.

A number of models have been proposed to explain odor identity and the associated valence in order to explain how a fly associates valence to a specific odor^73^. One approach, termed “labeled lines”, demonstrated that odors of particular relevance are often detected by ORNs expressing highly selective receptors that respond to only a single compound. In the case of *Drosophila*, this type of preferential relationship was demonstrated for sex pheromones, harmful substances, oviposition cues, and even food^74–78^. According to this model, activation of the labeled line generates a hardwired behavioral response. However, since most ORNs are broadly tuned to odors^37,68,79^, the labeled line approach is probably insufficient to explain behavioral responses in these cases. Indeed, behavioral responses to general odors are dependent on population neural activity^66^. Nevertheless, even under the population neural activity approach, a semi-labeled line assumption exists, according to which, a neuron always drives either attractive or aversive behavior (but not both), and it is the sum of the attractive and aversive signals from all the responding neurons that eventually determines the odor valence^66,67^. More recently, this notion was also demonstrated using optogenetic mapping of MBONs. Accordingly, an attractive or aversive behavior is observed following optogenetic activation of MBONs^24^, and appetitive or aversive associative conditioning will cause an odor to activate MBONs that drive attractive or aversive behavior, respectively^22,24^. This model is extremely suitable for the MB, where odor tuning of MBONs is modified by plasticity based on experience^23,25^. Recently, similar optogenetic mapping of LH neurons classified them as driving either attractive or aversive behavior^18^. However, it is well established that context affects innate behavioral output, which is mediated by the LH^31^. Thus, while the dynamic nature of KC-to-MBON connectivity allows for context-dependent changes, it is less clear how the LH, which as far as we know receives mostly hardwired inputs from PNs, makes context-dependent changes in neuronal activity (but see^15^). Our results suggest that at least for the LH, the notion that a neuron can be categorized as driving either attraction or aversion may need to be reconsidered.

## Methods

### Fly strains

Flies were grown on cornmeal agar under a 12 h light/12 h dark cycle at 25 °C. Experimental flies carrying transgenes were crossed with w^1118^ flies. The following transgenic flies were used: UAS-GCaMP6f^80^, UAS-Shibire^ts1^ ^81^, R37G11-GAL4^15^ (Bloomington #49539), R48F03-GAL4^21^ (Bloomington #50373), UAS-myrGFP,QUAS-mtdTomato(3XHA);*trans*-Tango^55^, MB011B, MB077B^24^ (Bloomington #68294, #68283, respectively). LH989^15,18^ was produced by combining R37G11-ZpGdbd and R29G05-p65ADZp promoter fragments. UAS-CD8::GFP, lexAop-rCD2::RFP^82^, nSyb-lexA.DBD::QF.AD (Bloomington #51953),UAS-DenMark^56^, UAS-^GFP^Dsyd-1^57^.

### Odors used

Odors were purchased from Sigma-Aldrich (Rehovot, Israel) and were at the purest level available. MA, methyl acetate; 2-but, 2-butanone; EL, ethyl lactate; EP, ethyl propionate; 2-pen, 2-pentanone; GA, geranyl acetate; AA, acetic acid; IAA, isoamyl acetate; HA, hexyl acetate; 2-hep, 2-heptanone; 3-oct, 3-octanol; 1-hex, 1-hexanol, g-dec, g-decalactone; MS, methyl salicylate; EB, ethyl benzoate; Benz, benzaldehyde; MCH, 4-methylcyclohexanol; Lin, linalool. ACV, apple cider vinegar was purchased locally (Rauch, Austria).

### Behavioral analysis

For the behavioral assay, 0–3 day old flies were placed in a fresh food vial and were tested at 7–14 days post-eclosion. When starved flies were used, they were placed in a fresh vial containing water-soaked filter paper 24 hours prior to the experiment.

As previously described, behavioral experiments were performed in an automated custom-behavior apparatus^54,60,61^. Single flies were placed in transparent chambers made of polycarbonate (dimensions 50*5*1.3 mm length*width*height). The chambers contained printed circuit boards (PCBs) on the floors and ceilings. PCBs were connected via Solid-state relays (Panasonic AQV253) to a 60 V source.

Mass flow controllers (CMOSens Performance Line, Sensirion) were used to control the air flow. Odors were streamed by passing the air flow through vials containing liquid odorant. A 0.3 l/min odor stream was combined with a 2.7 l/min air carried flow. Odors were diluted in mineral oil (Sigma-Aldrich, Rehovot), except ACV and acetic acid, which were diluted in DDW. Odors were prepared on a daily basis.

The total air flow (3 l/min) was split between 20 chambers. Thus each half chamber received a flow rate of 0.15 l/min. Odors were delivered to each half chamber using two identical odor delivery systems. The air flow from both sides of the chamber converged at the center of the chamber. Chambers were stacked in two columns, and were backlit by LEDs (940 nm, Vishay TSAL6400). A MAKO CMOS camera (Allied Vision Technologies) equipped with a Computar M0814-MP2 lens was used to obtain the images. The entire apparatus was placed in an incubator (Panasonic MIR 154) to control the temperature.

Each fly position over time was extracted from the video images using custom written software (LabVIEW 7.1, National Instruments) which also controlled the delivery of odors and electric shocks. Data were analyzed using custom written software (MATLAB 2015b, The MathWorks) and Prism 6 (GraphPad).

Preference was calculated as the percentage of time spent in one half of the chamber. Odor avoidance and training protocols were as presented in Fig. 2 and Supplementary Fig. S7. The naïve avoidance index was calculated as (preference for the left side when it contains odor) – (preference for the left side when it contains air). During training, an odor (usually the more attractive one) was paired with 12 equally spaced 1.25 s electric shocks at 60 V^49^. The learning index was calculated as (preference for odor before training) – (preference for odor after training).

A fly’s initial mean velocity was calculated from the first 3 seconds after the first odor pulse^47^. Velocity was calculated only for flies that were on the odor side upon odor presentation. Fly position was recorded every 175 ms and data was smoothed across 5 data points. Vectors of the fly’s heading were calculated based on fly position and were divided by 175 ms to deduce velocity. To calculate differences between odors, the integral of the response was calculated for each fly and averaged across flies. Mean velocity and integral were calculated using MATLAB 2015b (The MathWorks).

### Functional imaging

Flies used for functional imaging were reared as described above, but only 3–10 days post-eclosion females were used. A two-photon laser-scanning microscope^83,84^, DF-Scope installed on an Olympus BX51WI microscope) was used to image the brains. Flies were anesthetized by placing them on ice until they stopped moving and then a single fly was glued to aluminum foil and placed in a custom built chamber. The cuticle and trachea were removed, and the exposed brain was superfused with carbonated solution (95% O_2_, 5% CO_2_) containing 103 mM NaCl, 3 mM KCl, 5 mM trehalose, 10 mM glucose, 26 mM NaHCO_3_, 1 mM NaH_2_PO_4_, 1.5 mM CaCl_2_, 4 mM MgCl_2_, 5 mM N-Tris (TES), pH 7.3. Odors were delivered at a final concentration of 5×10^−2^ using mass-flow controllers (Sensirion) at a final flow rate of 0.8 l/min and controlled by solenoid valves (The Lee Company). Air-streamed odor was delivered through 1/16 inch ultra-chemical-resistant Versilon PVC tubing (Saint-Gobain, NJ, USA) that was placed 5 mm from the fly’s antenna. A Ti-Sapphire laser (Mai Tai HP DS, 100 fs pulses) centered at 910 nm was used to excite fluorescence. The laser was attenuated by a Pockels cell (Conoptics), coupled to a galvo-resonant scanner, and focused by a 20×, 1.0 NA objective (Olympus XLUMPLFLN20XW). The emitted photons were detected by GaAsP photomultiplier tubes (Hamamatsu Photonics, H10770PA-40SEL, Hamamatsu HC-130-INV). Images were captured using MScan 2.3.01.

For analysis, files were motion-corrected in X-Y using the TurboReg^85^ ImageJ plugin. ∆F/F and the inter-odor correlations were calculated as described previously^61^. Non-responsive flies and flies whose motion could not be corrected, were excluded.

Peak odor responses, area under curve, and time to peak were calculated by Prism (GraphPad) and were manually corrected where needed. Response persistence was calculated as the inverse of the ratio between peak odor responses and the average ∆F/F response 5 seconds after peak odor response was detected^86^. Responsiveness was calculated as the percentage of cells responding, out of the total number of cells examined for the specific odor.

### Structural imaging

Brain dissections, fixation, and immunostaining were performed as previously described^87,88^. In the case of native GFP and RFP fluorescence, brains were fixed at room temperature for 20 minutes in 4% (w/v) paraformaldehyde in PBS (1.86 mM NaH_2_PO_4_, 8.41 mM Na_2_HPO_4_, 175 mM NaCl). Samples were then washed three times for 20 minutes in PBS containing 0.3% (v/v) Triton-X-100 (PBT). The primary antisera used were mouse monoclonal anti-GFP IgG2a (1:500, Invitrogen), rabbit polyclonal anti-GFP (1:500, Invitrogen), mouse monoclonal anti-ChAT (1:100, 4B1, DSHB), rabbit polyclonal anti-dvGlut (1:10.000)^36^, and rabbit polyclonal anti-GABA (1:1.000, A2052, Sigma-Aldrich). Secondary antisera were Alexa488 coupled to goat anti-rabbit or goat anti-mouse IgG2a, Alexa647 coupled to goat anti-mouse (1:500, all Invitrogen), and STAR RED coupled to goat anti-rabbit (1:500, Abberior). Counterstaining was performed using nc82 (DSHB) and goat anti-mouse Alexa647 or Alexa564. Primary antisera were applied for 1–2 days and secondary antisera for 1–2 days in PBT at 4 °C, followed by embedding in Vectashield. Images were taken using a confocal microscope (Leica TCS SP5, SP8, Zeiss LSM 800, or a Nikon A1). Images were processed using ImageJ or Amira (ThermoFisher).

For *trans-*Tango experiments, R37G11-GAL4, MB077B, or MB011B males were crossed with UAS-myrGFP,QUAS-mtdTomato,*trans*-Tango virgin females and reared at 18 °C. Three-week-old flies were anesthetized using ice and then dissected and processed as described above.

### Correlation analysis

Correlation analysis was performed using MATLAB R2015a. Odor response vectors (∆F/F) were compared from the onset of odor response to the end of the recording (due to prolonged odor responses with some odors), 35 seconds later. To determine the within-fly correlation for a given odor vector, the odor responses of all neurons recorded (3–6 neurons) were compared. The averages of such correlation values for all odors and all flies were used. For odor correlation, all odor responses of a given neuron were compared. Similarly, the averages of such correlation values for all odors and all flies were used. To determine between-fly correlations, a matrix with all neurons from all flies for a given odor was constructed and all correlation values were calculated. An average correlation value was then calculated for each odor. The averages of such values for all odors were used. For control calculations, two approaches were used. First, a 3D matrix was composed as above but for all odors. Response vectors were then shuffled and the above calculation was repeated. We then repeated the calculation on an artificial data set with the same number of neurons, flies, and a correlation between the neurons of the same fly, but not between flies (the low odor correlation was used as the baseline for correlation).

### Statistics

All statistical analyses were performed using GraphPad Prism as described in the figure legends and in Table S1. In general, no statistical methods were used to predetermine the sample sizes.

## Supplementary information

Supplementary Information.

Main Figures source data.

Supplementary Figures source data.

## Data Availability

The datasets generated and analyzed for the current study are available from the corresponding author on request.
