# Supplementary material for: Differential Role for a Defined Lateral Horn Neuron Subset in Naïve Odor Valence in Drosophila
Source: Sci Rep. 2020 Apr 9;10:6147. doi: 10.1038/s41598-020-63169-3 (PMC7145822; doi:10.1038/s41598-020-63169-3)
Supplement: Supplementary file 1 — Supplementary Information. [file 41598_2020_63169_MOESM1_ESM.pdf]

Supplementary Information

Differential Role for a Defined Lateral Horn Neuron Subset in Naïve Odor Valence in *Drosophila*

Hadas Lerner, Eyal Rozenfeld, Bar Rozenman, Wolf Huetteroth, and Moshe Parnas

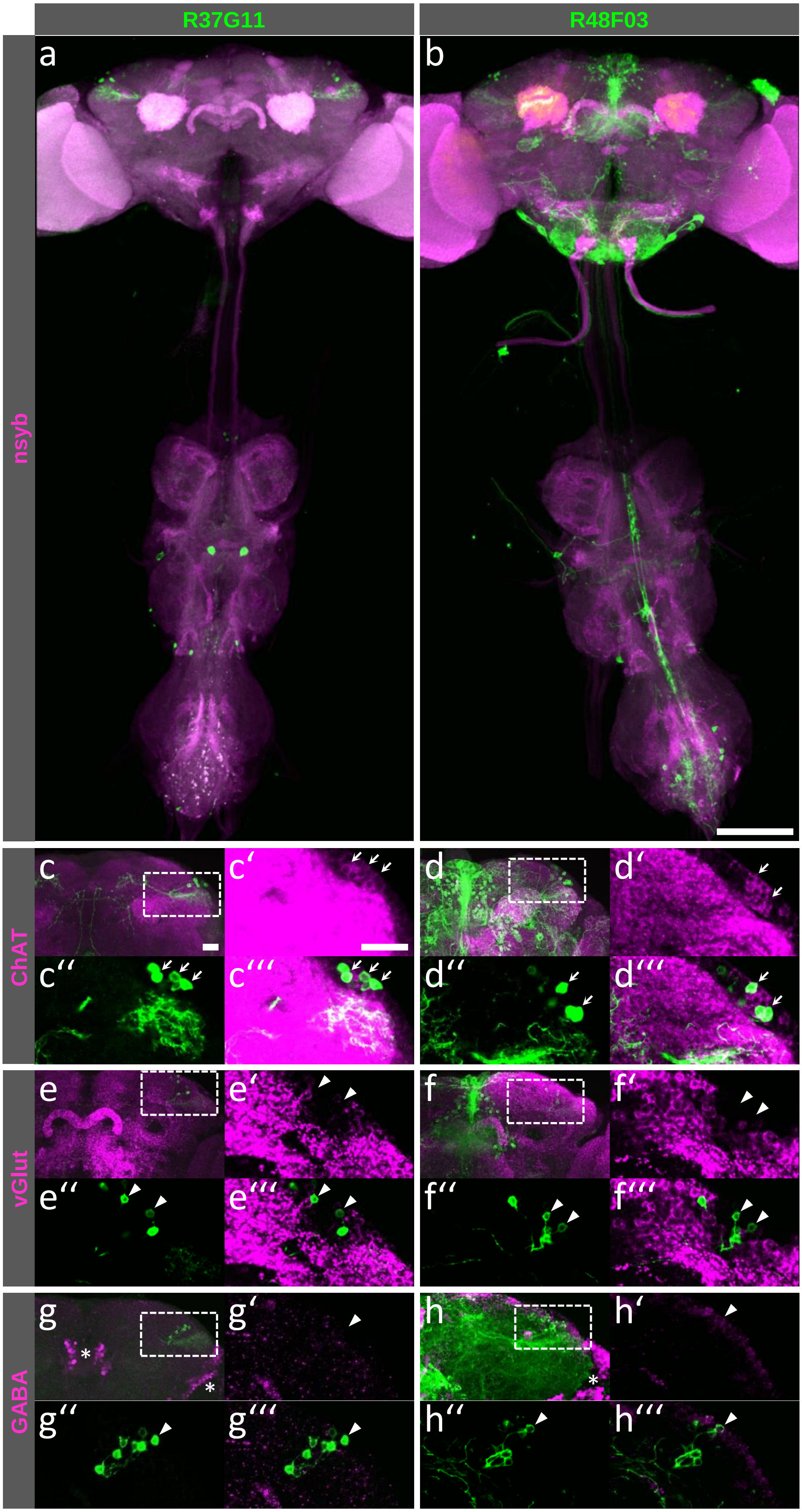

R37G11
R48F03
nsyb
ChAT
vGlut
GABA

Figure S1: R37G11-GAL4 and R48F03-GAL4 drivers both label cholinergic neurons in the LH

a. R37G11-GAL4 driven mCD8::GFP (green) shows a sparse label of 6-7 cells in the lateral horn and almost no label in the thoracico-abdominal ganglia (TAG). Neuropilar reference label by nsyb-driven rCD2::RFP (magenta).

b. R48F03-GAL4-driven mCD8::GFP (green) labels 18-22 cells in the lateral horn, but also shows staining in the pars intercerebralis, the fan-shaped body, the gnathal ganglia and the TAG. Neuropilar reference label was by nsyb-driven rCD2::RFP (magenta).

c-h'''. Immunohistochemical co-labeling of GFP (green) driven by both driver lines together with antisera against either acetylcholine-synthesizing enzyme ChAT (**c,d**), the glutamate transporter vGlut (**e,f**), or GABA **(g,h**) (magenta) reveals colocalization of ChAT in somata of both lines (arrows in **c'-d'''**), but not colocalization of vGlut or GABA (arrowheads in **e'-h'''**). GABA-positive cell groups can be seen in overviews (asterisks in **g, h**). Dashed boxes in overviews (**c-h**) denote the corresponding, same-lettered enlarged regions. **a-h**: projection views of 1,5 - 2 µm-thick optical sections; **c'-c''', d'-d''', e'-e''',f'-f''',g'-g''',h'-h'''**: single 1 µm-thick optical sections. Scale bars: **a,b**: 100 µm; **c-h'''**: 20 µm.

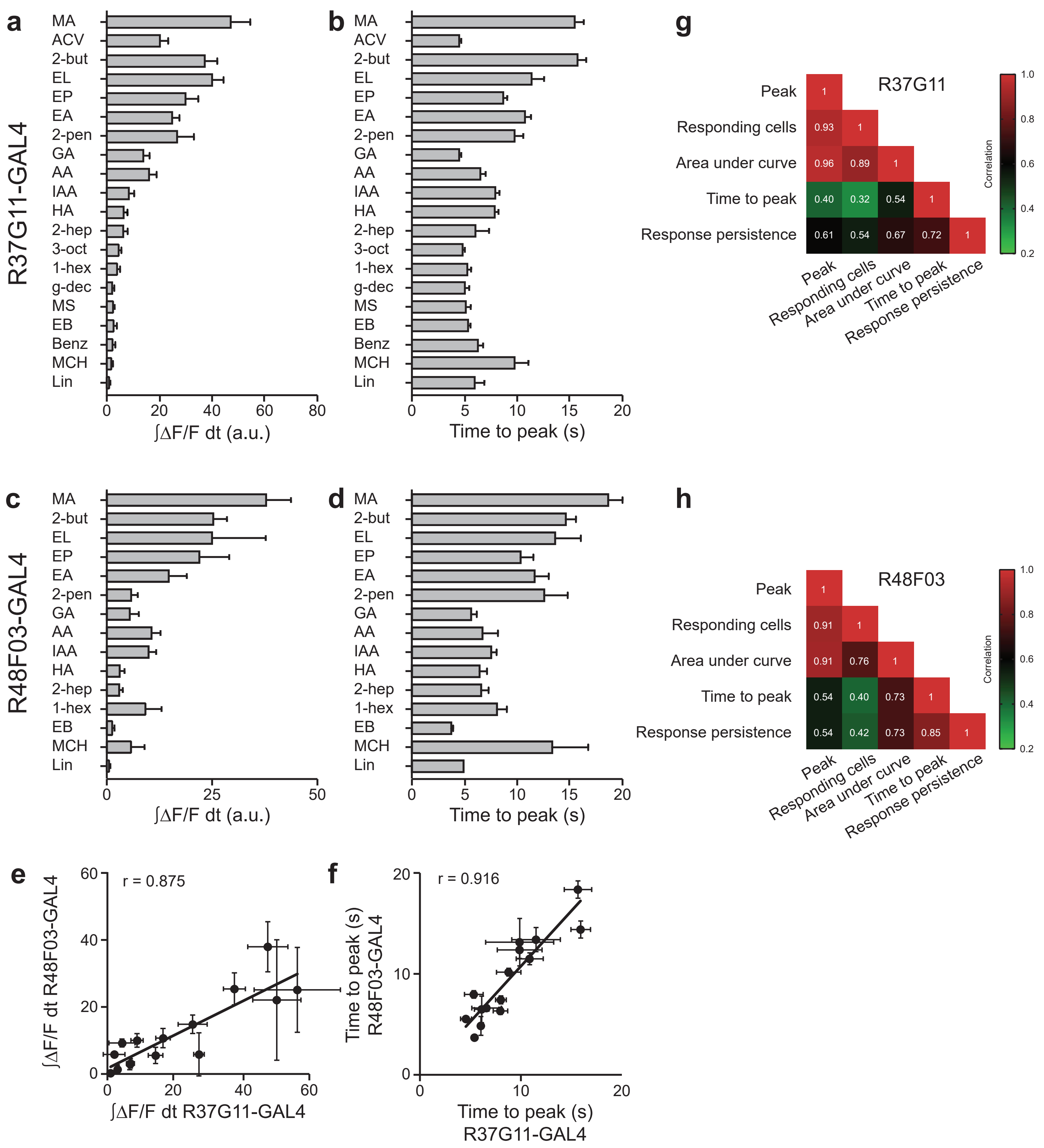
a
b
c
d
e
f
g
h
R37G11-GAL4
R48F03-GAL4
MA
ACV
2-but
EL
EP
EA
2-pen
GA
AA
IAA
HA
2-hep
3-oct
1-hex
g-dec
MS
EB
Benz
MCH
Lin
∫ΔF/F dt (a.u.)
Time to peak (s)
R37G11
R48F03
Peak
Responding cells
Area under curve
Time to peak
Response persistence
Correlation
r = 0.875
r = 0.916
∫ΔF/F dt R48F03-GAL4
∫ΔF/F dt R37G11-GAL4
Time to peak (s) R48F03-GAL4
Time to peak (s) R37G11-GAL4

Figure S2: PD2a1/b1 neurons display varied odor responses to high odor concentrations - Additional parameters for Fig 1

a, c. The integral of the odor response (from the onset of odor pulse and up to 35 s afterwards) for R37G11-GAL4 (a) and R48F03-GAL4 (c). ($55 \geq n \geq 20$ and $46 \geq n \geq 14$ cells, respectively. At least 5 different flies were imaged from each genotype.)

b, d. Time to peak analysis for R37G11-GAL4 (b) and R48F03-GAL4 (d). ($55 \geq n \geq 20$ and $46 \geq n \geq 14$ cells, respectively. At least 5 different flies were imaged from each genotype.)

e, f. Correlation between the parameters obtained for R37G11-GAL4 and R48F03-GAL4 for the integral of the odor response (e) and the time to peak (f).

g, h. Correlation values between peak responses, percent of responding neurons, response persistence, time to peak and the area under the curve was calculated for R37G11-GAL4 (g) and R48F03-GAL (h). (See Supplementary Table S1 for statistical analysis).

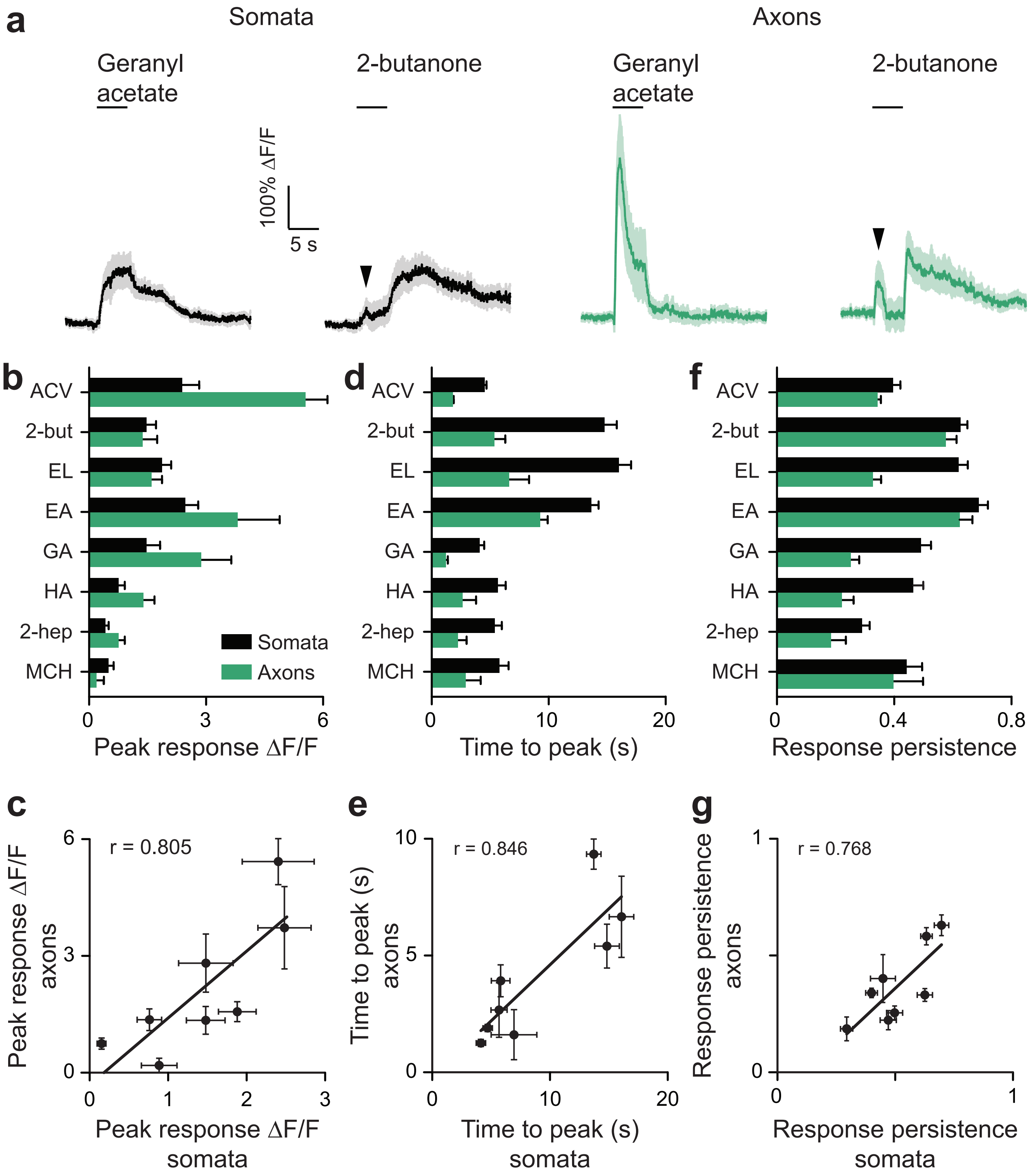
a
Somata
Axons
Geranyl acetate
2-butanone
100% ΔF/F
5 s
b
ACV
2-but
EL
EA
GA
HA
2-hep
MCH
Somata
Axons
Peak response ΔF/F
d
Time to peak (s)
f
Response persistence
c
r = 0.805
Peak response ΔF/F axons
Peak response ΔF/F somata
e
r = 0.846
Time to peak (s) axons
Time to peak (s) somata
g
r = 0.768
Response persistence axons
Response persistence somata

Figure S3: Odorant-evoked activity in PD2a1/b1 somata and axons

a. Examples of odor responses for somata (black) and axonal projections (green) for the designated odors. The black arrow indicates a fast transient response which is clearly observed at the axonal terminal and can still be observed, even though filtered, at the soma.

b. Analysis of the peak odor responses in somata and axonal projections. Axonal responses were significantly stronger than soma responses. ($47 \geq n \geq 24$ cells, from at least 5 different flies; see Supplementary Table S1 for statistical analysis.)

c. Correlation between peak responses obtained from somata and axonal projections. (See Supplementary Table S1 for statistical analysis.)

d. Analysis of the response time to the peak in somata and axonal projections. A significant reduction in response latency was observed at axonal projections ($47 \geq n \geq 24$ cells, from at least 5 different flies; see Supplementary Table S1 for statistical analysis.)

e. Correlation between the response time to the peak obtained at somata and axonal projections. Although responses at axonal projections are much faster, a high correlation was observed. (See Supplementary Table S1 for statistical analysis.)

f. Analysis of the response persistence in somata and axonal projections. The axonal responses decayed significantly faster than the soma responses. ($47 \geq n \geq 24$ cells, from at least 5 different flies; see Supplementary Table S1 for statistical analysis.)

g. Correlation between the response persistence in somata and axonal projections. (See Supplementary Table S1 for statistical analysis.)

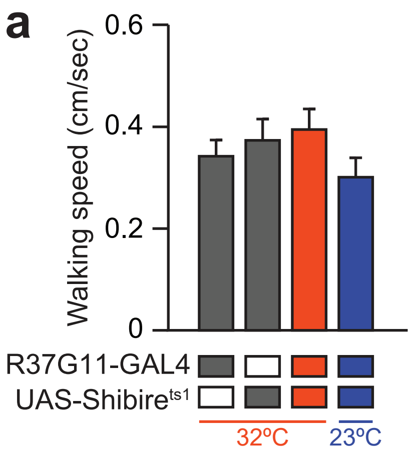

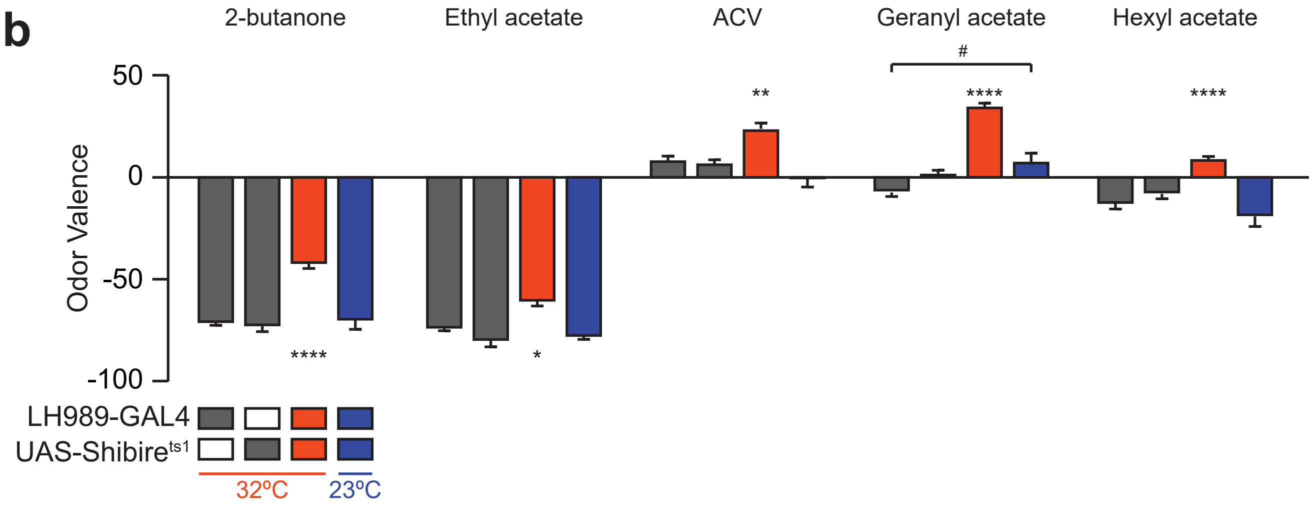

Figure S4: Silencing PD2a1/b1 LHN with the sparse split-GAL4 LH989 has similar results as obtained with R37G11-GAL4 – controls for Fig 3

a. Mean velocity scores during the 60 s prior to odor onset in the behavioral chamber. Gray, parental controls; red, the experimental group at 32 °C; blue, the experimental control group at 23 °C. No significant difference was observed. ($67 \geq n \geq 32$ flies; see Supplementary Table S1 for statistical analysis.)

b. Mean valence scores (see Methods) from experiments as in Fig. 3, for the designated odors using the sparser split-GAL4 driver line LH989. A consistent shift towards more positive valence values was observed during PD2a1/b1 inhibition ($79 \geq n \geq 25$ flies for all conditions, * indicates a significant difference of the indicated group from all other groups according to a multiple comparison test. The lowest value of all multiple comparison tests is presented. # indicates a significant difference only between the two indicated groups. *, # $p < 0.05$, ** $p < 0.01$, **** $p < 0.0001$, see Supplementary Table S1 for statistical analysis.)

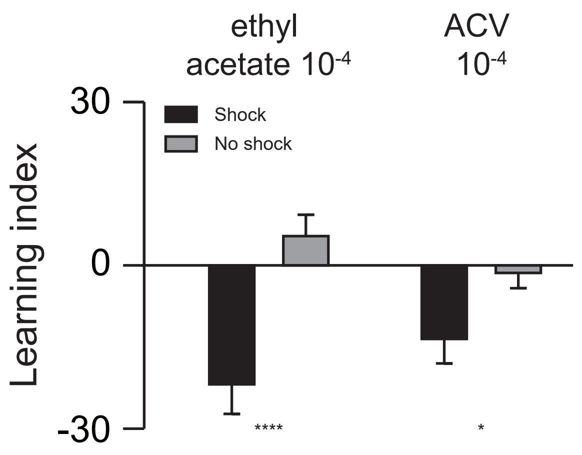

Figure S5: Flies perceive low odor concentration – control for Fig 4

Mean learning index (see Methods) for the designated odors. Flies show an aversion from both odors following conditioning. Control flies where mock training was used show no change in odor preference (66 ≥ n ≥ 45 flies, * $p < 0.05$, **** $p < 0.0001$; see Supplementary Table S1 for statistical analysis).

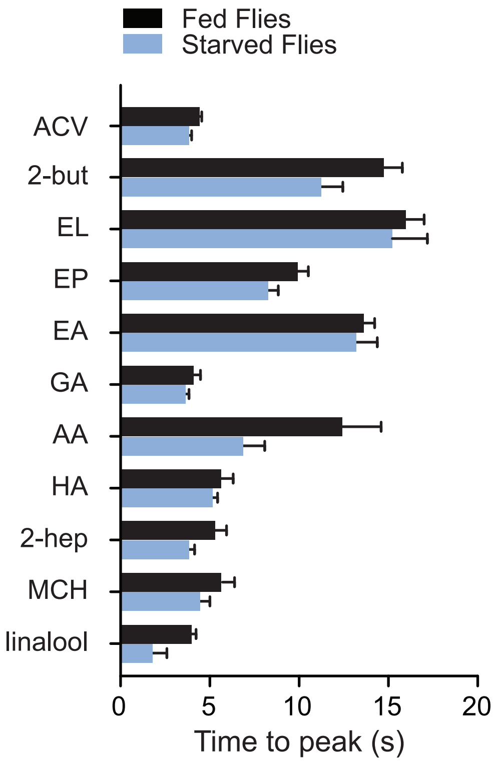

Figure S6: Starvation does not affect PD2a1/b1 odor responses. An additional parameter for Fig 5

Analysis of the time to peak of the response of the designated odors in fed (black) and starved (light blue) flies. A statistical difference was observed only with acetic acid (49 ≥ n ≥ 18 cells, from at least 5 different flies; see Supplementary Table S1 for statistical analysis.)

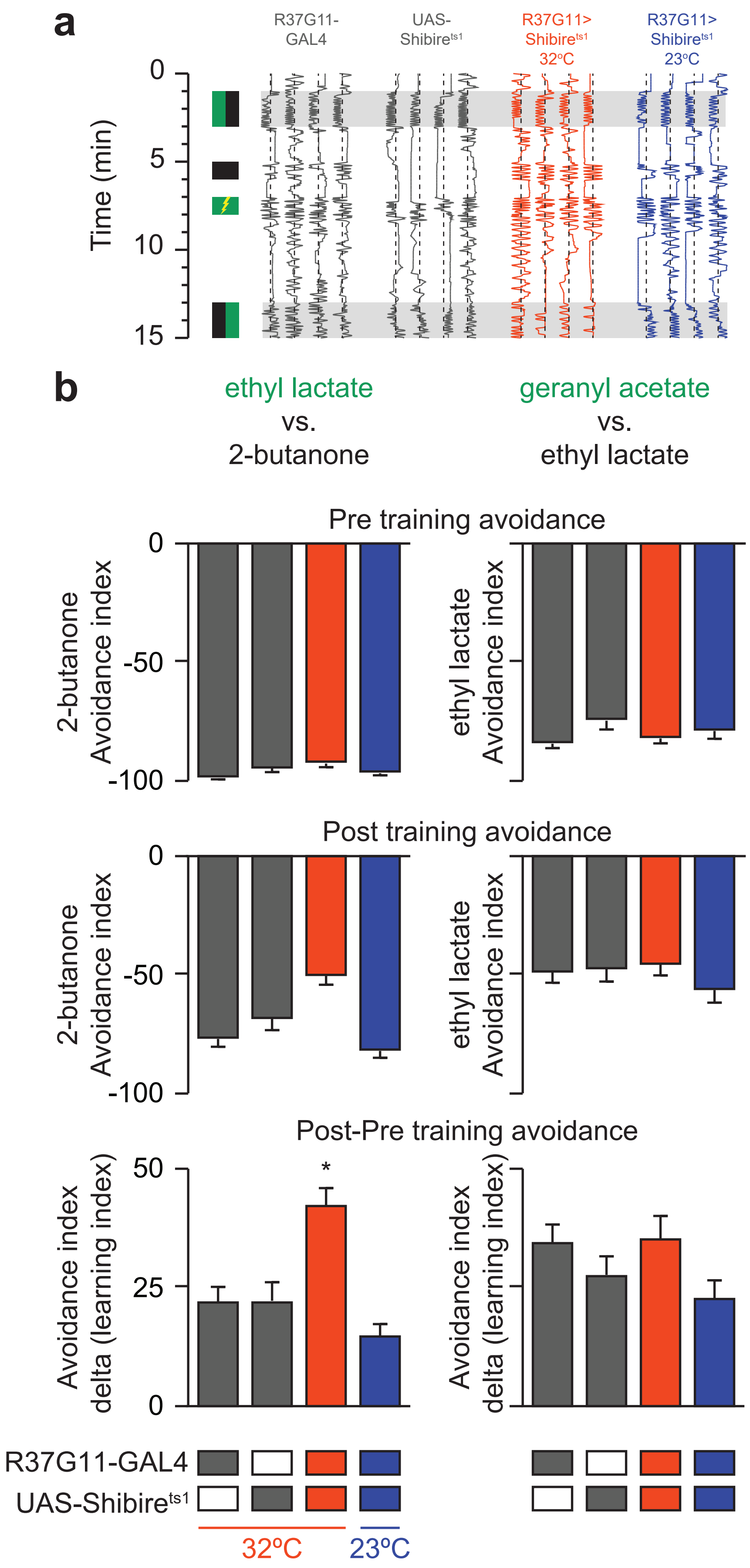
a
R37G11-GAL4
UAS-Shibire^{ts1}
R37G11>Shibire^{ts1} 32°C
R37G11>Shibire^{ts1} 23°C
Time (min)
0
5
10
15
b
ethyl lactate vs. 2-butanone
geranyl acetate vs. ethyl lactate
Pre training avoidance
2-butanone Avoidance index
ethyl lactate Avoidance index
0
-50
-100
Post training avoidance
Post-Pre training avoidance
Avoidance index delta (learning index)
50
25
*
R37G11-GAL4
UAS-Shibire^{ts1}
32°C
23°C

Figure S7: PD2a1/b1 neurons contribute to aversive conditioning at high odor concentrations

a. An example of traces obtained for conditioned flies. Ethyl lactate was used as the CS^+ and 2-butanone for the CS^-. Gray, parental controls; red, the experimental group at 32 °C; blue, the experimental control group at 23 °C. Dashed lines indicate the center of the behavior chamber. Lack of a shift in odor preference was observed for controls, whereas a shift in odor preference was observed for the experimental group.

b. Mean odor avoidance from experiments as in A for the CS^- odor (2-butanone in the 2-butanone/ethyl lactate pair and ethyl lactate in the ethyl lactate/geranyl acetate pair), before conditioning (*top),* following conditioning (middle), and the delta between the two measurements, also known as the learning index (bottom, see methods). Silencing PD2a1/b1 neurons before conditioning had no effect on the avoidance form the CS^- in these two odor pairs. However, when silencing PD2a1/b1 neurons reduced avoidance of the CS^-, 2-butanone, a large decrease in 2-butanone avoidance was observed following conditioning relative to parental and temperature controls. In the control experiment where silencing PD2a1/b1 neurons had no effect on the CS^-, ethyl lactate, no improvement in ethyl lactate avoidance was observed following conditioning relative to parental and temperature controls. ($92 \geq n \geq 33$ flies; * $p<0.05$; see Supplementary Table S1 for statistical analysis).

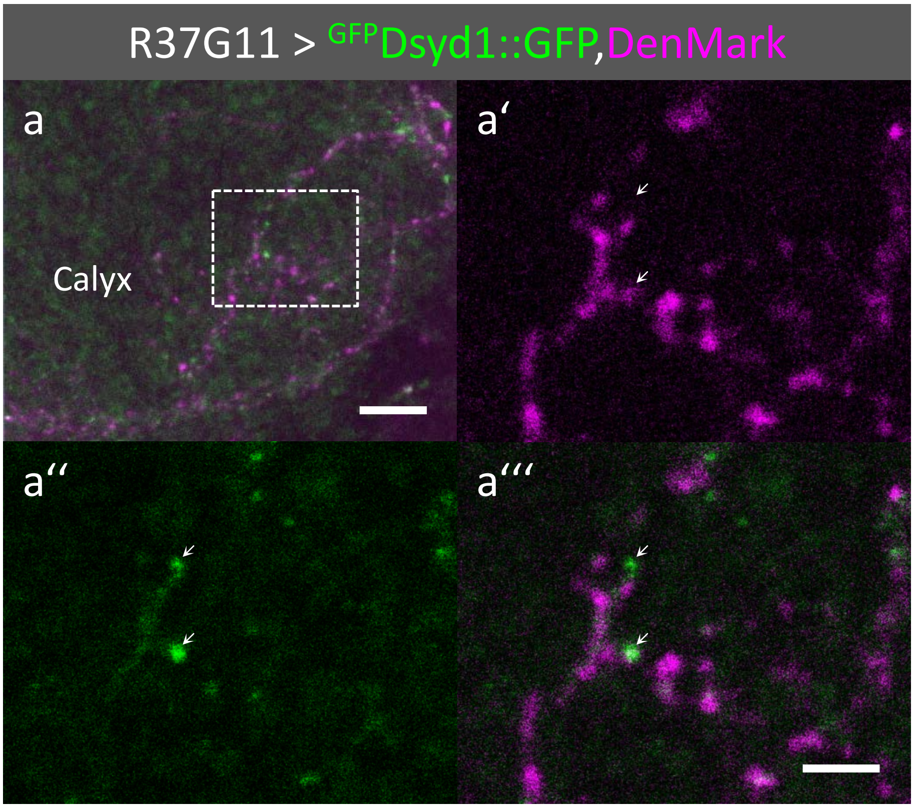
R37G11 > GFPDsyd1::GFP,DenMark
a
Calyx
a'
a''
a'''

Figure S8: PD2b1 neurites in the calyx display presynaptic sites

a: Projection view of the synaptic site marker ^{GFP}Dsyd-1 (green) and the dendritic marker DenMark (magenta) under the control of R37G11-GAL4 in the calyx. The dashed box denotes the corresponding, same-lettered enlarged region. **a'-a'''**: Single optical section (0.5 µm) shows putative presynaptic sites (arrows) of PD2b1 neurons in the calyx. Dendritic marker DenMark (**a'**, magenta), synaptic site marker ^{GFP}Dsyd-1 (**a''**, green) and overlay (**a'''**). Scale bars: **a**: 5 µm; **a'-a'''**: 2 µm.

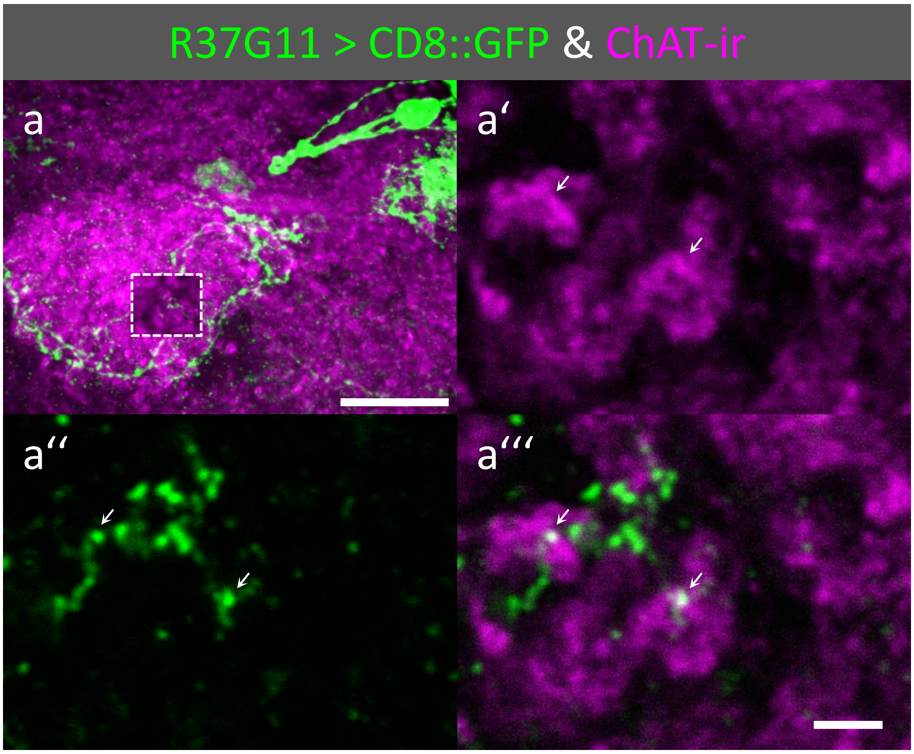
R37G11 > CD8::GFP & ChAT-ir
a
a'
a''
a'''

Figure S9: PD2b1 neurons in the calyx colocalize with ChAT-immunofluorescence

a: Projection view of CD8::GFP under the control of R37G11-GAL4 (green) and ChAT-ir (magenta) in the calyx. The dashed box denotes the corresponding, same-lettered enlarged region. **a'-a'''**: The single optical section (0.5 µm) reveals that ChAT-ir (**a'**, magenta) and R37G11-GAL4 neurites (**a''**, green) show overlap (**a'''**) in putative presynaptic sites (arrows) of PD2b1 neurons in the calyx. Scale bars: **a**: 20 µm; **a'-a'''**: 2 µm.

Table S1: Statistical analysis

Statistical analysis for the data presented in Figs. 1-6 and Supplementary Figs. S2-S7

Figure	Data	Statistical method	comparison	P-value or adjusted P-value	Significance
1K	Peak responses	Pearson correlation	1. R37G11-GAL4<br>2. R48F03-GAL4	<0.0001	****
1L	% responding cells	Pearson correlation	1. R37G11-GAL4<br>2. R48F03-GAL4	0.0019	**
1M	Response persistence	Pearson correlation	1. R37G11-GAL4<br>2. R48F03-GAL4	<0.0001	****
3B	2-Butanone	One-way non parametric ANOVA - Kruskal's-Wallis test	1. R37G11-GAL4<br>2. UAS-Shi^{ts1}<br>3. R37G11-GAL4> UAS-Shi^{ts1} 23˚<br>4. R37G11-GAL4> UAS-Shi^{ts1} 32°	<0.0001	****
		Dunn's multiple comparison test	1. R37G11-GAL4<br>2. UAS-Shi^{ts1}	0.608	N.S.
			1. R37G11-GAL4<br>2. R37G11-GAL4> UAS-Shi^{ts1} 23˚	>0.9999	N.S.
			1. UAS-Shi^{ts1}<br>2. R37G11-GAL4> UAS-Shi^{ts1} 23°	0.445	N.S.
			1. R37G11-GAL4<br>2. R37G11-GAL4> UAS-Shi^{ts1} 32°	<0.0001	****
			1. UAS-Shi^{ts1}<br>2. R37G11-GAL4> UAS-Shi^{ts1} 32°	<0.0001	****
			1. R37G11-GAL4> UAS-Shi^{ts1} 23°<br>2. R37G11-GAL4> UAS-Shi^{ts1} 32°	<0.0001	****
	Methyl Acetate	One-way non parametric ANOVA - Kruskal's-Wallis test	1. R37G11-GAL4<br>2. UAS-Shi^{ts1}<br>3. R37G11-GAL4> UAS-Shi^{ts1} 23°<br>4. R37G11-GAL4> UAS-Shi^{ts1} 32°	<0.0001	****
		Dunn's multiple comparison test	1. R37G11-GAL4<br>2. UAS-Shi^{ts1}	0.024	*
			1. R37G11-GAL4<br>2. R37G11-GAL4> UAS-Shi^{ts1} 23°	0.146	N.S.
			1. UAS-Shi^{ts1}<br>2. R37G11-GAL4> UAS-Shi^{ts1} 23°	>0.999	N.S.
			1. R37G11-GAL4<br>2. R37G11-GAL4> UAS-Shi^{ts1} 32°	0.028	*
			1. UAS-Shi^{ts1}<br>2. R37G11-GAL4> UAS-Shi^{ts1} 32°	<0.0001	****
			1. R37G11-GAL4> UAS-Shi^{ts1} 23°<br>2. R37G11-GAL4> UAS-Shi^{ts1} 32°	<0.0001	****
	Ethyl Acetate	One-way non parametric ANOVA - Kruskal's-Wallis test	1. R37G11-GAL4<br>2. UAS-Shi^{ts1}<br>3. R37G11-GAL4> UAS-Shi^{ts1} 23°<br>4. R37G11-GAL4> UAS-Shi^{ts1} 32°	<0.0001	****
		Dunn's multiple comparison test	1. R37G11-GAL4<br>2. UAS-Shi^{ts1}	0.255	N.S.
			1. R37G11-GAL4<br>2. R37G11-GAL4> UAS-Shi^{ts1} 23°	0.003	**
			1. UAS-Shi^{ts1}<br>2. R37G11-GAL4> UAS-Shi^{ts1} 23°	>0.999	N.S.
			1. R37G11-GAL4	0.048	*

		2. R37G11-GAL4> UAS-Shi^{ts1} 32°		
		1. UAS-Shi^{ts1}<br>2. R37G11-GAL4> UAS-Shi^{ts1} 32°	<0.0001	****
		1. R37G11-GAL4> UAS-Shi^{ts1} 23°<br>2. R37G11-GAL4> UAS-Shi^{ts1} 32°	<0.0001	****
Ethyl Lactate	One-way non parametric ANOVA - Kruskal's-Wallis test	1. R37G11-GAL4<br>2. UAS-Shi^{ts1}<br>3. R37G11-GAL4> UAS-Shi^{ts1} 23°<br>4. R37G11-GAL4> UAS-Shi^{ts1} 32°	0.04	*
	Dunn's multiple comparison test	1. R37G11-GAL4<br>2. UAS-Shi^{ts1}	0.259	N.S.
		1. R37G11-GAL4<br>2. R37G11-GAL4> UAS-Shi^{ts1} 23°	>0.999	N.S.
		1. UAS-Shi^{ts1}<br>2. R37G11-GAL4> UAS-Shi^{ts1} 23°	>0.999	N.S.
		1. R37G11-GAL4<br>2. R37G11-GAL4> UAS-Shi^{ts1} 32°	>0.999	N.S.
		1. UAS-Shi^{ts1}<br>2. R37G11-GAL4> UAS-Shi^{ts1} 32°	0.035	*
		1. R37G11-GAL4> UAS-Shi^{ts1} 23°<br>2. R37G11-GAL4> UAS-Shi^{ts1} 32°	>0.999	N.S.
2-Heptanone	One-way non parametric ANOVA - Kruskal's-Wallis test	1. R37G11-GAL4<br>2. UAS-Shi^{ts1}<br>3. R37G11-GAL4> UAS-Shi^{ts1} 23°<br>4. R37G11-GAL4> UAS-Shi^{ts1} 32°	0.0001	***
	Dunn's multiple comparison test	1. R37G11-GAL4<br>2. UAS-Shi^{ts1}	0.446	N.S.
		1. R37G11-GAL4<br>2. R37G11-GAL4> UAS-Shi^{ts1} 23°	>0.999	N.S.
		1. UAS-Shi^{ts1}<br>2. R37G11-GAL4> UAS-Shi^{ts1} 23°	>0.999	N.S.
		1. R37G11-GAL4<br>2. R37G11-GAL4> UAS-Shi^{ts} 32°	0.027	*
		1. UAS-Shi^{ts1}<br>2. R37G11-GAL4> UAS-Shi^{ts1} 32°	<0.0001	****
		1. R37G11-GAL4> UAS-Shi^{ts1} 23°<br>2. R37G11-GAL4> UAS-Shi^{ts1} 32°	0.018	*
Hexyl Acetate	One-way non parametric ANOVA - Kruskal's-Wallis test	1. R37G11-GAL4<br>2. UAS-Shi^{ts1}<br>3. R37G11-GAL4> UAS-Shi^{ts1} 23°<br>4. R37G11-GAL4> UAS-Shi^{ts1} 32°	<0.0001	****
	Dunn's multiple comparison test	1. R37G11-GAL4<br>2. UAS-Shi^{ts1}	0.522	N.S.
		1. R37G11-GAL4<br>2. R37G11-GAL4> UAS- Shi^{ts1} 23°	>0.999	N.S.
		1. UAS- Shi^{ts1}<br>2. R37G11-GAL4> UAS- Shi^{ts1} 23°	>0.999	N.S.
		1. R37G11-GAL4<br>2. R37G11-GAL4> UAS- Shi^{ts1} 32°	<0.0001	****
		1. UAS- Shi^{ts1}<br>2. R37G11-GAL4> UAS- Shi^{ts1} 32°	0.002	**
		1. R37G11-GAL4> UAS- Shi^{ts1} 23°<br>2. R37G11-GAL4> UAS- Shi^{ts1} 32°	<0.0001	****
Geranyl acetate	One-way non parametric ANOVA - Kruskal's-Wallis test	1. R37G11-GAL4<br>2. UAS- Shi^{ts1}<br>3. R37G11-GAL4> UAS- Shi^{ts1} 23°<br>4. R37G11-GAL4> UAS- Shi^{ts1} 32°	<0.0001	****

	Dunn's multiple comparison test	1. R37G11-GAL4<br>2. UAS- Shi^{ts1}	>0.999	N.S.
		1. R37G11-GAL4<br>2. R37G11-GAL4> UAS- Shi^{ts1} 23°	>0.999	N.S.
		1. UAS- Shi^{ts1}<br>2. R37G11-GAL4> UAS- Shi^{ts1} 23°	0.337	N.S.
		1. R37G11-GAL4<br>2. R37G11-GAL4> UAS- Shi^{ts1} 32°	<0.0001	****
		1. UAS- Shi^{ts1}<br>2. R37G11-GAL4> UAS- Shi^{ts1} 32°	<0.0001	****
		1. R37G11-GAL4> UAS- Shi^{ts1} 23°<br>2. R37G11-GAL4> UAS- Shi^{ts1} 32°	0.006	**
ACV	One-way non parametric ANOVA - Kruskal's-Wallis test	1. R37G11-GAL4<br>2. UAS- Shi^{ts1}<br>3. R37G11-GAL4> UAS- Shi^{ts1} 23°<br>4. R37G11-GAL4> UAS- Shi^{ts1} 32°	<0.0001	****
	Dunn's multiple comparison test	1. R37G11-GAL4<br>2. UAS- Shi^{ts1}	>0.999	N.S.
		1. R37G11-GAL4<br>2. R37G11-GAL4> UAS- Shi^{ts1} 23°	0.056	N.S.
		1. UAS- Shi^{ts1}<br>2. R37G11-GAL4> UAS- Shi^{ts1} 23°	0.825	N.S.
		1. R37G11-GAL4<br>2. R37G11-GAL4> UAS- Shi^{ts1} 32°	<0.0001	****
		1. UAS- Shi^{ts1}<br>2. R37G11-GAL4> UAS- Shi^{ts1} 32°	0.001	**
		1. R37G11-GAL4> UAS- Shi^{ts1} 23°<br>2. R37G11-GAL4> UAS- Shi^{ts1} 32°	<0.0001	****
MCH	One-way non parametric ANOVA - Kruskal's-Wallis test	1. R37G11-GAL4<br>2. UAS- Shi^{ts1}<br>3. R37G11-GAL4> UAS- Shi^{ts1} 23°<br>4. R37G11-GAL4> UAS- Shi^{ts1} 32°	<0.0001	****
	Dunn's multiple comparison test	1. R37G11-GAL4<br>2. UAS- Shi^{ts1}	>0.999	N.S.
		1. R37G11-GAL4<br>2. R37G11-GAL4> UAS- Shi^{ts1} 23°	<0.0001	****
		1. UAS- Shi^{ts1}<br>2. R37G11-GAL4> UAS- Shi^{ts1} 23°	0.001	**
		1. R37G11-GAL4<br>2. R37G11-GAL4> UAS- Shi^{ts1} 32°	>0.999	N.S.
		1. UAS- Shi^{ts1}<br>2. R37G11-GAL4> UAS- Shi^{ts1} 32°	>0.999	N.S.
		1. R37G11-GAL4> UAS- Shi^{ts1} 23°<br>2. R37G11-GAL4> UAS- Shi^{ts1} 32°	0.0003	***
3-Octanol	One-way non parametric ANOVA - Kruskal's-Wallis test	1. R37G11-GAL4<br>2. UAS- Shi^{ts1}<br>3. R37G11-GAL4> UAS-Shi^{ts1} 23°<br>4. R37G11-GAL4> UAS-Shi^{ts1} 32°	<0.0001	****
	Dunn's multiple comparison test	1. R37G11-GAL4<br>2. UAS- Shi^{ts1}	>0.999	N.S.
		1. R37G11-GAL4<br>2. R37G11-GAL4> UAS- Shi^{ts1} 23°	<0.0001	****
		1. UAS- Shi^{ts1}<br>2. R37G11-GAL4> UAS- Shi^{ts1} 23°	0.0004	***
		1. R37G11-GAL4<br>2. R37G11-GAL4> UAS- Shi^{ts1} 32°	>0.999	N.S.
		1. UAS- Shi^{ts1}	0.21	N.S.

			2. R37G11-GAL4> UAS- Shi^{ts1} 32°		
			1. R37G11-GAL4> UAS- Shi^{ts1} 23°<br>2. R37G11-GAL4> UAS- Shi^{ts1} 32°	<0.0001	****
	Ethyl benzoate	One-way non parametric ANOVA - Kruskal's-Wallis test	1. R37G11-GAL4<br>2. UAS- Shi^{ts1}<br>3. R37G11-GAL4> UAS- Shi^{ts1} 23°<br>4. R37G11-GAL4> UAS- Shi^{ts1} 32°	0.002	**
		Dunn's multiple comparison test	1. R37G11-GAL4<br>2. UAS- Shi^{ts1}	>0.999	N.S.
			1. R37G11-GAL4<br>2. R37G11-GAL4> UAS- Shi^{ts1} 23°	0.196	N.S.
			1. UAS- Shi^{ts1}<br>2. R37G11-GAL4> UAS- Shi^{ts1} 23°	0.013	*
			1. R37G11-GAL4<br>2. R37G11-GAL4> UAS- Shi^{ts1} 32°	0.717	N.S.
			1. UAS- Shi^{ts1}<br>2. R37G11-GAL4> UAS- Shi^{ts1} 32°	>0.999	N.S.
			1. R37G11-GAL4> UAS- Shi^{ts1} 23°<br>2. R37G11-GAL4> UAS- Shi^{ts1} 32°	0.002	**
	1-Hexanol	One-way non parametric ANOVA - Kruskal's-Wallis test	1. R37G11-GAL4<br>2. UAS- Shi^{ts1}<br>3. R37G11-GAL4> UAS- Shi^{ts1} 23°<br>4. R37G11-GAL4> UAS- Shi^{ts1} 32°	0.0002	***
		Dunn's multiple comparison test	1. R37G11-GAL4<br>2. UAS- Shi^{ts1}	0.0004	***
			1. R37G11-GAL4<br>2. R37G11-GAL4> UAS- Shi^{ts1} 23°	>0.999	N.S.
			1. UAS- Shi^{ts1}<br>2. R37G11-GAL4> UAS- Shi^{ts1} 23°	0.003	**
			1. R37G11-GAL4<br>2. R37G11-GAL4> UAS- Shi^{ts1} 32°	0.067	N.S.
			1. UAS- Shi^{ts1}<br>2. R37G11-GAL4> UAS- Shi^{ts1} 32°	0.49	N.S.
			1. R37G11-GAL4> UAS- Shi^{ts1} 23°<br>2. R37G11-GAL4> UAS- Shi^{ts1} 32°	0.318	N.S.
4B	Peak response ΔF/F	Multiple t-test with Sidak-Bonferroni correction	1. HA 1X10-2<br>2. HA 5X10-2	0.43	N.S.
			1. 2-hep 1X10-2<br>2. 2-hep 5X10-2	0.04	*
			1. GA 1X10-2<br>2. GA 5X10-2	0.55	N.S
			1. EA 1X10-2<br>2. EA 5X10-2	0.08	N.S.
			1. ACV 1X10-2<br>2. ACV 5X10-2	0.003	**
			1. 2-butanone 1X10-2<br>2. 2-butanone 5X10-2	0.24	N.S.
4C	2-butanone 10-3	One-way non parametric ANOVA - Kruskal's-Wallis test	1. R37G11-GAL4<br>2. UAS- Shi^{ts1}<br>3. R37G11-GAL4> UAS- Shi^{ts1} 23°<br>4. R37G11-GAL4> UAS- Shi^{ts1} 32°	<0.0001	****
		Dunn's multiple comparison test	1. R37G11-GAL4<br>2. UAS- Shi^{ts1}	0.03	*
			1. R37G11-GAL4<br>2. R37G11-GAL4> UAS- Shi^{ts1} 23°	<0.0001	****
			1. UAS- Shi^{ts1}<br>2. R37G11-GAL4> UAS- Shi^{ts1} 23°	0.1	N.S.

		1. R37G11-GAL4<br>2. R37G11-GAL4> UAS- Shi^{ts1} 32°	0.02	*
		1. UAS- Shi^{ts1}<br>2. R37G11-GAL4> UAS- Shi^{ts1} 32°	>0.999	N.S.
		1. R37G11-GAL4> UAS- Shi^{ts1} 23°<br>2. R37G11-GAL4> UAS- Shi^{ts1} 32°	0.19	N.S.
2-butanone 10-4	One-way non parametric ANOVA - Kruskal's-Wallis test	1. R37G11-GAL4<br>2. UAS- Shi^{ts1}<br>3. R37G11-GAL4> UAS- Shi^{ts1} 23°<br>4. R37G11-GAL4> UAS- Shi^{ts1} 32°	0.01	*
	Dunn's multiple comparison test	1. R37G11-GAL4<br>2. UAS- Shi^{ts1}	>0.999	N.S.
		1. R37G11-GAL4<br>2. R37G11-GAL4> UAS- Shi^{ts1} 23°	0.01	*
		1. UAS- Shi^{ts1}<br>2. R37G11-GAL4> UAS- Shi^{ts1} 23°	0.04	*
		1. R37G11-GAL4<br>2. R37G11-GAL4> UAS- Shi^{ts1} 32°	0.46	N.S.
		1. UAS- Shi^{ts1}<br>2. R37G11-GAL4> UAS- Shi^{ts1} 32°	>0.999	N.S.
		1. R37G11-GAL4> UAS- Shi^{ts1} 23°<br>2. R37G11-GAL4> UAS- Shi^{ts1} 32°	0.66	N.S.
Ethyl acetate 10-3	One-way non parametric ANOVA - Kruskal's-Wallis test	1. R37G11-GAL4<br>2. UAS- Shi^{ts1}<br>3. R37G11-GAL4> UAS- Shi^{ts1} 23°<br>4. R37G11-GAL4> UAS- Shi^{ts1} 32°	0.002	**
	Dunn's multiple comparison test	1. R37G11-GAL4<br>2. UAS- Shi^{ts1}	>0.999	N.S.
		1. R37G11-GAL4<br>2. R37G11-GAL4> UAS- Shi^{ts1} 23°	0.09	N.S.
		1. UAS- Shi^{ts1}<br>2. R37G11-GAL4> UAS- Shi^{ts1} 23°	0.23	N.S.
		1. R37G11-GAL4<br>2. R37G11-GAL4> UAS- Shi^{ts1} 32°	>0.999	N.S.
		1. UAS- Shi^{ts1}<br>2. R37G11-GAL4> UAS- Shi^{ts1} 32°	0.5	N.S.
		1. R37G11-GAL4> UAS- Shi^{ts1} 23°<br>2. R37G11-GAL4> UAS- Shi^{ts1} 32°	0.001	***
Ethyl acetate 10-4	One-way non parametric ANOVA - Kruskal's-Wallis test	1. R37G11-GAL4<br>2. UAS- Shi^{ts1}<br>3. R37G11-GAL4> UAS- Shi^{ts1} 23°<br>4. R37G11-GAL4> UAS- Shi^{ts1} 32°	<0.0001	****
	Dunn's multiple comparison test	1. R37G11-GAL4<br>2. UAS- Shi^{ts1}	0.29	N.S.
		1. R37G11-GAL4<br>2. R37G11-GAL4> UAS- Shi^{ts1} 23°	0.055	N.S.
		1. UAS- Shi^{ts1}<br>2. R37G11-GAL4> UAS- Shi^{ts1} 23°	<0.0001	****
		1. R37G11-GAL4<br>2. R37G11-GAL4> UAS- Shi^{ts1} 32°	>0.999	N.S.
		1. UAS- Shi^{ts1}<br>2. R37G11-GAL4> UAS- Shi^{ts1} 32°	0.22	N.S.
		1. R37G11-GAL4> UAS- Shi^{ts1} 23°<br>2. R37G11-GAL4> UAS- Shi^{ts1} 32°	0.02	*
Geranyl acetate 10-3	One-way non parametric ANOVA - Kruskal's-Wallis test	1. R37G11-GAL4<br>2. UAS- Shi^{ts1}<br>3. R37G11-GAL4> UAS- Shi^{ts1} 23°<br>4. R37G11-GAL4> UAS- Shi^{ts1} 32°	0.84	N.S.

	Geranyl acetate 10-4	One-way non parametric ANOVA - Kruskal's-Wallis test	1. R37G11-GAL4<br>2. UAS- Shi^{ts1}<br>3. R37G11-GAL4> UAS- Shi^{ts1} 23°<br>4. R37G11-GAL4> UAS- Shi^{ts1} 32°	0.007	**
		Dunn's multiple comparison test	1. R37G11-GAL4<br>2. UAS- Shi^{ts1}	0.07	N.S.
			1. R37G11-GAL4<br>2. R37G11-GAL4> UAS- Shi^{ts1} 23°	>0.999	N.S.
			1. UAS- Shi^{ts1}<br>2. R37G11-GAL4> UAS- Shi^{ts1} 23°	0.02	*
			1. R37G11-GAL4<br>2. R37G11-GAL4> UAS- Shi^{ts1} 32°	0.44	N.S.
			1. UAS- Shi^{ts1}<br>2. R37G11-GAL4> UAS- Shi^{ts1} 32°	>0.999	N.S.
			1. R37G11-GAL4> UAS- Shi^{ts1} 23°<br>2. R37G11-GAL4> UAS- Shi^{ts1} 32°	0.16	N.S.
	ACV 10-3	One-way non parametric ANOVA - Kruskal's-Wallis test	1. R37G11-GAL4<br>2. UAS- Shi^{ts1}<br>3. R37G11-GAL4> UAS- Shi^{ts1} 23°<br>4. R37G11-GAL4> UAS- Shi^{ts1} 32°	0.0003	***
		Dunn's multiple comparison test	1. R37G11-GAL4<br>2. UAS- Shi^{ts1}	>0.999	N.S.
			1. R37G11-GAL4<br>2. R37G11-GAL4> UAS- Shi^{ts1} 23°	>0.999	N.S.
			1. UAS- Shi^{ts1}<br>2. R37G11-GAL4> UAS- Shi^{ts1} 23°	>0.999	N.S.
			1. R37G11-GAL4<br>2. R37G11-GAL4> UAS- Shi^{ts1} 32°	0.02	*
			1. UAS- Shi^{ts1}<br>2. R37G11-GAL4> UAS- Shi^{ts1} 32°	0.002	**
			1. R37G11-GAL4> UAS- Shi^{ts1} 23°<br>2. R37G11-GAL4> UAS- Shi^{ts1} 32°	0.002	**
	ACV 10-4	One-way non parametric ANOVA - Kruskal's-Wallis test	1. R37G11-GAL4<br>2. UAS- Shi^{ts1}<br>3. R37G11-GAL4> UAS- Shi^{ts1} 23°<br>4. R37G11-GAL4> UAS- Shi^{ts1} 32°	0.002	**
		Dunn's multiple comparison test	1. R37G11-GAL4<br>2. UAS- Shi^{ts1}	>0.999	N.S.
			1. R37G11-GAL4<br>2. R37G11-GAL4> UAS- Shi^{ts1} 23°	0.01	*
			1. UAS- Shi^{ts1}<br>2. R37G11-GAL4> UAS- Shi^{ts1} 23°	0.005	**
			1. R37G11-GAL4<br>2. R37G11-GAL4> UAS- Shi^{ts1} 32°	>0.999	N.S.
			1. UAS- Shi^{ts1}<br>2. R37G11-GAL4> UAS- Shi^{ts1} 32°	>0.999	N.S.
			1. R37G11-GAL4> UAS- Shi^{ts1} 23°<br>2. R37G11-GAL4> UAS- Shi^{ts1} 32°	0.01	*
4D	Peak response ΔF/F Vs. |Δodor valence|	Pearson correlation	1. Peak response ΔF/F<br>2. |Δodor valence|	0.36	N.S.
5B	Peak responses	Two-way ordinary ANOVA	Interaction	0.31	N.S.
			Main effect of odor	<0.0001	****

	Responses persistence	Two-way ordinary ANOVA	Main effect of condition (Fed Vs starved)	0.35	N.S.
			Interaction	0.0006	***
			Main effect of odor	<0.0001	****
			Main effect of condition (Fed Vs starved)	0.71	N.S.
5C	2-Heptanone	One-way non parametric ANOVA - Kruskal's-Wallis test	1. R37G11-GAL4<br>2. UAS- Shi^{ts1}<br>3. R37G11-GAL4> UAS- Shi^{ts1} 23°<br>4. R37G11-GAL4> UAS- Shi^{ts1} 32°	<0.0001	****
		Dunn's multiple comparison test	1. R37G11-GAL4<br>2. UAS- Shi^{ts1}	0.866	N.S.
			1. R37G11-GAL4<br>2. R37G11-GAL4> UAS- Shi^{ts1} 23°	0.0001	***
			1. UAS- Shi^{ts1}<br>2. R37G11-GAL4> UAS- Shi^{ts1} 23°	<0.0001	****
			1. R37G11-GAL4<br>2. R37G11-GAL4> UAS- Shi^{ts1} 32°	>0.999	N.S.
			1. UAS- Shi^{ts1}<br>2. R37G11-GAL4> UAS- Shi^{ts1} 32°	>0.999	N.S.
			1. R37G11-GAL4> UAS- Shi^{ts1} 23°<br>2. R37G11-GAL4> UAS- Shi^{ts1} 32°	<0.0001	****
	Hexyl Acetate	One-way non parametric ANOVA - Kruskal's-Wallis test	1. R37G11-GAL4<br>2. UAS- Shi^{ts1}<br>3. R37G11-GAL4> UAS- Shi^{ts1} 23°<br>4. R37G11-GAL4> UAS- Shi^{ts1} 32°	0.054	N.S.
	ACV	One-way non parametric ANOVA - Kruskal's-Wallis test	1. R37G11-GAL4<br>2. UAS- Shi^{ts1}<br>3. R37G11-GAL4> UAS- Shi^{ts1} 23°<br>4. R37G11-GAL4> UAS- Shi^{ts1} 32°	0.18	N.S.
	Geranyl acetate	One-way non parametric ANOVA - Kruskal's-Wallis test	1. R37G11-GAL4<br>2. UAS- Shi^{ts1}<br>3. R37G11-GAL4> UAS- Shi^{ts1} 23°<br>4. R37G11-GAL4> UAS- Shi^{ts1} 32°	0.008	**
		Dunn's multiple comparison test	1. R37G11-GAL4<br>2. UAS- Shi^{ts1}	0.51	N.S.
			1. R37G11-GAL4<br>2. R37G11-GAL4> UAS- Shi^{ts1} 23°	>0.999	N.S.
			1. UAS- Shi^{ts1}<br>2. R37G11-GAL4> UAS- Shi^{ts1} 23°	0.01	*
			1. R37G11-GAL4<br>2. R37G11-GAL4> UAS- Shi^{ts1} 32°	>0.999	N.S.
			1. UAS- Shi^{ts1}<br>2. R37G11-GAL4> UAS- Shi^{ts1} 32°	0.03	*
			1. R37G11-GAL4> UAS- Shi^{ts1} 23°<br>2. R37G11-GAL4> UAS- Shi^{ts1} 32°	>0.999	N.S.
	2-butanone	One-way non parametric ANOVA - Kruskal's-Wallis test	1. R37G11-GAL4<br>2. UAS- Shi^{ts1}<br>3. R37G11-GAL4> UAS- Shi^{ts1} 23°<br>4. R37G11-GAL4> UAS- Shi^{ts1} 32°	<0.0001	****
		Dunn's multiple comparison test	1. R37G11-GAL4<br>2. UAS- Shi^{ts1}	0.21	N.S.
			1. R37G11-GAL4<br>2. R37G11-GAL4> UAS- Shi^{ts1} 23°	0.009	**

			1. UAS- Shi^{ts1}<br>2. R37G11-GAL4> UAS- Shi^{ts1} 23°	<0.0001	****
			1. R37G11-GAL4<br>2. R37G11-GAL4> UAS- Shi^{ts1} 32°	0.005	**
			1. UAS- Shi^{ts1}<br>2. R37G11-GAL4> UAS- Shi^{ts1} 32°	<0.0001	****
			1. R37G11-GAL4> UAS- Shi^{ts1} 23°<br>2. R37G11-GAL4> UAS- Shi^{ts1} 32°	>0.999	N.S.
	Ethyl acetate	One-way non parametric ANOVA - Kruskal's-Wallis test	1. R37G11-GAL4<br>2. UAS- Shi^{ts1}<br>3. R37G11-GAL4> UAS- Shi^{ts1} 23°<br>4. R37G11-GAL4> UAS- Shi^{ts1} 32°	<0.0001	****
		Dunn's multiple comparison test	1. R37G11-GAL4<br>2. UAS- Shi^{ts1}	0.07	N.S.
			1. R37G11-GAL4<br>2. R37G11-GAL4> UAS- Shi^{ts1} 23°	0.0019	**
			1. UAS- Shi^{ts1}<br>2. R37G11-GAL4> UAS- Shi^{ts1} 23°	<0.0001	****
			1. R37G11-GAL4<br>2. R37G11-GAL4> UAS- Shi^{ts1} 32°	>0.999	N.S.
			1. UAS- Shi^{ts1}<br>2. R37G11-GAL4> UAS- Shi^{ts1} 32°	0.06	N.S.
			1. R37G11-GAL4> UAS- Shi^{ts1} 23°<br>2. R37G11-GAL4> UAS- Shi^{ts1} 32°	0.002	**
	Methyl acetate	One-way non parametric ANOVA - Kruskal's-Wallis test	1. R37G11-GAL4<br>2. UAS- Shi^{ts1}<br>3. R37G11-GAL4> UAS- Shi^{ts1} 23°<br>4. R37G11-GAL4> UAS- Shi^{ts1} 32°	<0.0001	****
		Dunn's multiple comparison test	1. R37G11-GAL4<br>2. UAS- Shi^{ts1}	0.003	**
			1. R37G11-GAL4<br>2. R37G11-GAL4> UAS- Shi^{ts1} 23°	<0.0001	****
			1. UAS-Shi^{ts1}<br>2. R37G11-GAL4> UAS- Shi^{ts1} 23°	<0.0001	****
			1. R37G11-GAL4<br>2. R37G11-GAL4> UAS- Shi^{ts1} 32°	0.009	**
			1. UAS- Shi^{ts1}<br>2. R37G11-GAL4> UAS- Shi^{ts1} 32°	<0.0001	****
			1. R37G11-GAL4> UAS- Shi^{ts1} 23°<br>2. R37G11-GAL4> UAS- Shi^{ts1} 32°	>0.999	N.S.
6C	Area Under the Curve	One-way non parametric ANOVA - Kruskal's-Wallis test	1. Geranyl acetate<br>2. ACV<br>3. Ethyl acetate<br>4. 2-heptanone	<0.0001	****
		Dunn's multiple comparison test	1. GA<br>2. ACV	>0.999	N.S.
			1. GA<br>2. EA	0.008	**
			1. GA<br>2. 2-hep	0.0006	***
			1. ACV<br>2. EA	0.009	**
			1. ACV<br>2. 2-hep	0.0006	***
			1. EA<br>2. 2-hep	>0.999	N.S.
6D	ACV	One-way non parametric ANOVA -	1. R37G11-GAL4	0.01	*

		Kruskal's-Wallis test	2. UAS- Shi^{ts1}<br>3. R37G11-GAL4> UAS- Shi^{ts1} 23°<br>4. R37G11-GAL4> UAS- Shi^{ts1} 32°		
		Dunn's multiple comparison test	1. R37G11-GAL4<br>2. UAS- Shi^{ts1}	0.44	N.S.
			1. R37G11-GAL4<br>2. R37G11-GAL4> UAS- Shi^{ts1} 23°	>0.999	N.S.
			1. UAS- Shi^{ts1}<br>2. R37G11-GAL4> UAS- Shi^{ts1} 23°	0.17	N.S.
			1. R37G11-GAL4<br>2. R37G11-GAL4> UAS- Shi^{ts1} 32°	>0.999	N.S.
			1. UAS- Shi^{ts1}<br>2. R37G11-GAL4> UAS- Shi^{ts1} 32°	0.01	*
			1. R37G11-GAL4> UAS- Shi^{ts1} 23°<br>2. R37G11-GAL4> UAS- Shi^{ts1} 32°	>0.999	N.S.
	Geranyl acetate	One-way non parametric ANOVA - Kruskal's-Wallis test	1. R37G11-GAL4<br>2. UAS- Shi^{ts1}<br>3. R37G11-GAL4> UAS- Shi^{ts1} 23°<br>4. R37G11-GAL4> UAS- Shi^{ts1} 32°	0.12	N.S.
	2-butanone	One-way non parametric ANOVA - Kruskal's-Wallis test	1. R37G11-GAL4<br>2. UAS- Shi^{ts1}<br>3. R37G11-GAL4> UAS- Shi^{ts1} 23°<br>4. R37G11-GAL4> UAS- Shi^{ts1} 32°	0.19	N.S.
	2-heptanone	One-way non parametric ANOVA - Kruskal's-Wallis test	1. R37G11-GAL4<br>2. UAS- Shi^{ts1}<br>3. R37G11-GAL4> UAS- Shi^{ts1} 23°<br>4. R37G11-GAL4> UAS- Shi^{ts1} 32°	0.11	N.S.
	Hexyl acetate	One-way non parametric ANOVA - Kruskal's-Wallis test	1. R37G11-GAL4<br>2. UAS- Shi^{ts1}<br>3. R37G11-GAL4> UAS- Shi^{ts1} 23°<br>4. R37G11-GAL4> UAS- Shi^{ts1} 32°	0.84	N.S.
	Ethyl acetate	One-way non parametric ANOVA - Kruskal's-Wallis test	1. R37G11-GAL4<br>2. UAS- Shi^{ts1}<br>3. R37G11-GAL4> UAS- Shi^{ts1} 23°<br>4. R37G11-GAL4> UAS- Shi^{ts1} 32°	0.1	N.S.
	Methyl acetate	One-way non parametric ANOVA - Kruskal's-Wallis test	1. R37G11-GAL4<br>2. UAS- Shi^{ts1}<br>3. R37G11-GAL4> UAS- Shi^{ts1} 23°<br>4. R37G11-GAL4> UAS- Shi^{ts1} 32°	0.81	N.S.
6E	2-butanone 40 sec	One-way non parametric ANOVA - Kruskal's-Wallis test	1. R37G11-GAL4<br>2. UAS- Shi^{ts1}<br>3. R37G11-GAL4> UAS- Shi^{ts1} 23°<br>4. R37G11-GAL4> UAS- Shi^{ts1} 32°	<0.0001	N.S.
		Dunn's multiple comparison test	1. R37G11-GAL4<br>2. UAS- Shi^{ts1}	0.055	N.S.
			1. R37G11-GAL4<br>2. R37G11-GAL4> UAS- Shi^{ts1} 23°	0.41	N.S.
			1. UAS-Shi^{ts1}<br>2. R37G11-GAL4> UAS-Shi^{ts1} 23°	0.0003	***
			1. R37G11-GAL4<br>2. R37G11-GAL4> UAS- Shi^{ts1} 32°	<0.0001	****
			1. UAS- Shi^{ts1}<br>2. R37G11-GAL4> UAS- Shi^{ts1} 32°	<0.0001	****
			1. R37G11-GAL4> UAS- Shi^{ts1} 23°<br>2. R37G11-GAL4> UAS- Shi^{ts1} 32°	0.22	N.S.

2-butanone<br>80 sec	One-way non parametric ANOVA - Kruskal's-Wallis test	1. R37G11-GAL4<br>2. UAS- Shi^{ts1}<br>3. R37G11-GAL4> UAS- Shi^{ts1} 23°<br>4. R37G11-GAL4> UAS- Shi^{ts1} 32°	<0.0001	****
	Dunn's multiple comparison test	1. R37G11-GAL4<br>2. UAS- Shi^{ts1}	>0.999	N.S.
		1. R37G11-GAL4<br>2. R37G11-GAL4> UAS- Shi^{ts1} 23°	>0.999	N.S.
		1. UAS- Shi^{ts1}<br>2. R37G11-GAL4> UAS- Shi^{ts1} 23°	0.477	N.S.
		1. R37G11-GAL4<br>2. R37G11-GAL4> UAS- Shi^{ts1} 32°	<0.0001	****
		1. UAS- Shi^{ts1}<br>2. R37G11-GAL4> UAS- Shi^{ts1} 32°	<0.0001	****
		1. R37G11-GAL4> UAS- Shi^{ts1} 23°<br>2. R37G11-GAL4> UAS- Shi^{ts1} 32°	0.003	**
Methyl acetate<br>40 sec	One-way non parametric ANOVA - Kruskal's-Wallis test	1. R37G11-GAL4<br>2. UAS- Shi^{ts1}<br>3. R37G11-GAL4> UAS- Shi^{ts1} 23°<br>4. R37G11-GAL4> UAS- Shi^{ts1} 32°	0.01	*
	Dunn's multiple comparison test	1. R37G11-GAL4<br>2. UAS- Shi^{ts1}	0.12	N.S.
		1. R37G11-GAL4<br>2. R37G11-GAL4> UAS- Shi^{ts1} 23°	0.08	N.S.
		1. UAS- Shi^{ts1}<br>2. R37G11-GAL4> UAS- Shi^{ts1} 23°	>0.999	N.S.
		1. R37G11-GAL4<br>2. R37G11-GAL4> UAS- Shi^{ts1} 32°	>0.999	N.S.
		1. UAS- Shi^{ts1}<br>2. R37G11-GAL4> UAS- Shi^{ts1} 32°	0.31	N.S.
		1. R37G11-GAL4> UAS- Shi^{ts1} 23°<br>2. R37G11-GAL4> UAS- Shi^{ts1} 32°	0.16	N.S.
Methyl acetate<br>80 sec	One-way non parametric ANOVA - Kruskal's-Wallis test	1. R37G11-GAL4<br>2. UAS- Shi^{ts1}<br>3. R37G11-GAL4> UAS- Shi^{ts1} 23°<br>4. R37G11-GAL4> UAS- Shi^{ts1} 32°	<0.0001	****
	Dunn's multiple comparison test	1. R37G11-GAL4<br>2. UAS- Shi^{ts1}	<0.0001	****
		1. R37G11-GAL4<br>2. R37G11-GAL4> UAS- Shi^{ts1} 23°	0.0002	***
		1. UAS- Shi^{ts1}<br>2. R37G11-GAL4> UAS- Shi^{ts1} 23°	>0.999	N.S.
		1. R37G11-GAL4<br>2. R37G11-GAL4> UAS- Shi^{ts1} 32°	>0.999	N.S.
		1. UAS- Shi^{ts1}<br>2. R37G11-GAL4> UAS- Shi^{ts1} 32°	<0.0001	****
		1. R37G11-GAL4> UAS- Shi^{ts1} 23°<br>2. R37G11-GAL4> UAS- Shi^{ts1} 32°	0.0004	***
Ethyl acetate<br>40 sec	One-way non parametric ANOVA - Kruskal's-Wallis test	1. R37G11-GAL4<br>2. UAS- Shi^{ts1}<br>3. R37G11-GAL4> UAS- Shi^{ts1} 23°<br>4. R37G11-GAL4> UAS- Shi^{ts1} 32°	<0.0001	****
	Dunn's multiple comparison test	1. R37G11-GAL4<br>2. UAS- Shi^{ts1}	0.03	*
		1. R37G11-GAL4<br>2. R37G11-GAL4> UAS- Shi^{ts1} 23°	0.0002	***
		1. UAS- Shi^{ts1}<br>2. R37G11-GAL4> UAS- Shi^{ts1} 23°	0.94	N.S.
		3. R37G11-GAL4	>0.999	N.S.

		4. R37G11-GAL4> UAS- Shi^{ts1} 32°		
		1. UAS- Shi^{ts1}<br>2. R37G11-GAL4> UAS- Shi^{ts1} 32°	0.03	*
		1. R37G11-GAL4> UAS- Shi^{ts1} 23°<br>2. R37G11-GAL4> UAS- Shi^{ts1} 32°	0.0002	***
Ethyl acetate 80 sec	One-way non parametric ANOVA - Kruskal's-Wallis test	1. R37G11-GAL4<br>2. UAS- Shi^{ts1}<br>3. R37G11-GAL4> UAS- Shi^{ts1} 23°<br>4. R37G11-GAL4> UAS- Shi^{ts1} 32°	<0.0001	****
	Dunn's multiple comparison test	1. R37G11-GAL4<br>2. UAS- Shi^{ts1}	<0.0001	****
		1. R37G11-GAL4<br>2. R37G11-GAL4> UAS- Shi^{ts1} 23°	<0.0001	****
		1. UAS- Shi^{ts1}<br>2. R37G11-GAL4> UAS- Shi^{ts1} 23°	>0.999	N.S.
		1. R37G11-GAL4<br>2. R37G11-GAL4> UAS- Shi^{ts1} 32°	>0.999	N.S.
		1. UAS- Shi^{ts1}<br>2. R37G11-GAL4> UAS- Shi^{ts1} 32°	0.0002	***
		1. R37G11-GAL4> UAS- Shi^{ts1} 23°<br>2. R37G11-GAL4> UAS- Shi^{ts1} 32°	<0.0001	****
2-heptanone 40 sec	One-way non parametric ANOVA - Kruskal's-Wallis test	1. R37G11-GAL4<br>2. UAS- Shi^{ts1}<br>3. R37G11-GAL4> UAS- Shi^{ts1} 23°<br>4. R37G11-GAL4> UAS- Shi^{ts1} 32°	<0.0001	****
	Dunn's multiple comparison test	1. R37G11-GAL4<br>2. UAS- Shi^{ts1}	0.01	*
		1. R37G11-GAL4<br>2. R37G11-GAL4> UAS- Shi^{ts1} 23°	0.13	N.S.
		1. UAS- Shi^{ts1}<br>2. R37G11-GAL4> UAS- Shi^{ts1} 23°	>0.999	N.S.
		1. R37G11-GAL4<br>2. R37G11-GAL4> UAS- Shi^{ts1} 32°	0.62	N.S.
		1. UAS- Shi^{ts1}<br>2. R37G11-GAL4> UAS- Shi^{ts1} 32°	<0.001	****
		1. R37G11-GAL4> UAS- Shi^{ts1} 23°<br>2. R37G11-GAL4> UAS- Shi^{ts1} 32°	0.001	**
2-heptanone 80 sec	One-way non parametric ANOVA - Kruskal's-Wallis test	1. R37G11-GAL4<br>2. UAS- Shi^{ts1}<br>3. R37G11-GAL4> UAS- Shi^{ts1} 23°<br>4. R37G11-GAL4> UAS- Shi^{ts1} 32°	0.0003	***
	Dunn's multiple comparison test	1. R37G11-GAL4<br>2. UAS- Shi^{ts1}	0.002	**
		1. R37G11-GAL4<br>2. R37G11-GAL4> UAS- Shi^{ts1} 23°	0.88	N.S.
		1. UAS- Shi^{ts1}<br>2. R37G11-GAL4> UAS- Shi^{ts1} 23°	0.55	N.S.
		1. R37G11-GAL4<br>2. R37G11-GAL4> UAS- Shi^{ts1} 32°	>0.999	N.S.
		1. UAS- Shi^{ts1}<br>2. R37G11-GAL4> UAS- Shi^{ts1} 32°	0.0007	***
		1. R37G11-GAL4> UAS- Shi^{ts1} 23°<br>2. R37G11-GAL4> UAS- Shi^{ts1} 32°	0.34	N.S.
Hexyl acetate 40 sec	One-way non parametric ANOVA - Kruskal's-Wallis test	1. R37G11-GAL4<br>2. UAS- Shi^{ts1}<br>3. R37G11-GAL4> UAS- Shi^{ts1} 23°<br>4. R37G11-GAL4> UAS- Shi^{ts1} 32°	0.02	*

	Dunn's multiple comparison test	1. R37G11-GAL4<br>2. UAS- Shi^{ts1}	0.54	N.S.
		1. R37G11-GAL4<br>2. R37G11-GAL4> UAS- Shi^{ts1} 23°	>0.999	N.S.
		1. UAS- Shi^{ts1}<br>2. R37G11-GAL4> UAS- Shi^{ts1} 23°	0.9	N.S.
		1. R37G11-GAL4<br>2. R37G11-GAL4> UAS- Shi^{ts1} 32°	0.06	N.S.
		1. UAS- Shi^{ts1}<br>2. R37G11-GAL4> UAS- Shi^{ts1} 32°	>0.999	N.S.
		1. R37G11-GAL4> UAS- Shi^{ts1} 23°<br>2. R37G11-GAL4> UAS- Shi^{ts1} 32°	0.09	N.S.
Hexyl acetate 80 sec	One-way non parametric ANOVA - Kruskal's-Wallis test	1. R37G11-GAL4<br>2. UAS- Shi^{ts1}<br>3. R37G11-GAL4> UAS- Shi^{ts1} 23°<br>4. R37G11-GAL4> UAS- Shi^{ts1} 32°	0.0001	***
	Dunn's multiple comparison test	1. R37G11-GAL4<br>2. UAS- Shi^{ts1}	0.66	N.S.
		1. R37G11-GAL4<br>2. R37G11-GAL4> UAS- Shi^{ts1} 23°	>0.999	N.S.
		1. UAS- Shi^{ts1}<br>2. R37G11-GAL4> UAS- Shi^{ts1} 23°	>0.999	N.S.
		1. R37G11-GAL4<br>2. R37G11-GAL4> UAS- Shi^{ts1} 32°	0.0006	***
		1. UAS- Shi^{ts1}<br>2. R37G11-GAL4> UAS- Shi^{ts1} 32°	0.03	*
		1. R37G11-GAL4> UAS- Shi^{ts1} 23°<br>2. R37G11-GAL4> UAS- Shi^{ts1} 32°	0.0009	***
Geranyl acetate 40 sec	One-way non parametric ANOVA - Kruskal's-Wallis test	1. R37G11-GAL4<br>2. UAS- Shi^{ts1}<br>3. R37G11-GAL4> UAS- Shi^{ts1} 23°<br>4. R37G11-GAL4> UAS- Shi^{ts1} 32°	0.0001	***
	Dunn's multiple comparison test	1. R37G11-GAL4<br>2. UAS- Shi^{ts1}	>0.999	N.S.
		1. R37G11-GAL4<br>2. R37G11-GAL4> UAS- Shi^{ts1} 23°	0.1	N.S.
		1. UAS- Shi^{ts1}<br>2. R37G11-GAL4> UAS- Shi^{ts1} 23°	0.29	N.S.
		1. R37G11-GAL4<br>2. R37G11-GAL4> UAS- Shi^{ts1} 32°	0.006	***
		1. UAS- Shi^{ts1}<br>2. R37G11-GAL4> UAS- Shi^{ts1} 32°	0.003	**
		1. R37G11-GAL4> UAS- Shi^{ts1} 23°<br>2. R37G11-GAL4> UAS- Shi^{ts1} 32°	>0.999	N.S.
Geranyl acetate 80 sec	One-way non parametric ANOVA - Kruskal's-Wallis test	1. R37G11-GAL4<br>2. UAS- Shi^{ts1}<br>3. R37G11-GAL4> UAS- Shi^{ts1} 23°<br>4. R37G11-GAL4> UAS- Shi^{ts1} 32°	<0.0001	****
	Dunn's multiple comparison test	1. R37G11-GAL4<br>2. UAS- Shi^{ts1}	>0.999	N.S.
		1. R37G11-GAL4<br>2. R37G11-GAL4> UAS- Shi^{ts1} 23°	0.24	N.S.
		1. UAS- Shi^{ts1}<br>2. R37G11-GAL4> UAS- Shi^{ts1} 23°	0.23	N.S.
		1. R37G11-GAL4<br>2. R37G11-GAL4> UAS- Shi^{ts1} 32°	<0.001	****
		1. UAS- Shi^{ts1}<br>2. R37G11-GAL4> UAS- Shi^{ts1} 32°	<0.0001	****

			1. R37G11-GAL4> UAS- Shi^{ts1} 23°<br>2. R37G11-GAL4> UAS- Shi^{ts1} 32°	0.04	*
	ACV 40 sec	One-way non parametric ANOVA - Kruskal's-Wallis test	1. R37G11-GAL4<br>2. UAS- Shi^{ts1}<br>3. R37G11-GAL4> UAS- Shi^{ts1} 23°<br>4. R37G11-GAL4> UAS- Shi^{ts1} 32°	0.0008	***
		Dunn's multiple comparison test	1. R37G11-GAL4<br>2. UAS- Shi^{ts1}	>0.999	N.S.
			1. R37G11-GAL4<br>2. R37G11-GAL4> UAS- Shi^{ts1} 23°	>0.999	N.S.
			1. UAS- Shi^{ts1}<br>2. R37G11-GAL4> UAS- Shi^{ts1} 23°	0.59	N.S.
			1. R37G11-GAL4<br>2. R37G11-GAL4> UAS- Shi^{ts1} 32°	0.006	**
			1. UAS- Shi^{ts1}<br>2. R37G11-GAL4> UAS- Shi^{ts1} 32°	>0.999	N.S.
			1. R37G11-GAL4> UAS- Shi^{ts1} 23°<br>2. R37G11-GAL4> UAS- Shi^{ts1} 32°	0.002	**
	ACV 80 sec	One-way non parametric ANOVA - Kruskal's-Wallis test	1. R37G11-GAL4<br>2. UAS- Shi^{ts1}<br>3. R37G11-GAL4> UAS- Shi^{ts1} 23°<br>4. R37G11-GAL4> UAS- Shi^{ts1} 32°	<0.0001	****
		Dunn's multiple comparison test	1. R37G11-GAL4<br>2. UAS- Shi^{ts1}	>0.999	N.S.
			1. R37G11-GAL4<br>2. R37G11-GAL4> UAS-Shi^{ts1} 23°	0.08	N.S.
			1. UAS-Shi^{ts1}<br>2. R37G11-GAL4> UAS- Shi^{ts1} 23°	0.09	N.S.
			1. R37G11-GAL4<br>2. R37G11-GAL4> UAS- Shi^{ts1} 32°	0.07	N.S.
			1. UAS- Shi^{ts1}<br>2. R37G11-GAL4> UAS- Shi^{ts1} 32°	0.65	N.S.
			1. R37G11-GAL4> UAS- Shi^{ts1} 23°<br>2. R37G11-GAL4> UAS- Shi^{ts1} 32°	<0.0001	****
S2E	$\int \Delta F/F\, dt$	Pearson correlation	1. R37G11-GAL4<br>2. R48F03-GAL4	<0.0001	****
S2F	Time to peak (s)	Pearson correlation	1. R37G11-GAL4<br>2. R48F03-GAL4	<0.0001	****
S2G	R37G11	Spearman correlation (non-parametric)	1. Peak<br>2. Percent responding cells	<0.0001	****
			1. Peak<br>2. Area under curve	<0.0001	****
			1. Peak<br>2. Time to peak	0.076	N.S.
			1. Peak<br>2. Response persistence	0.004	**
			1. Percent responding cells<br>2. Area under curve	<0.0001	****
			1. Percent responding cells<br>2. Time to peak	0.156	N.S.
			1. Percent responding cells<br>2. Response persistence	0.013	*
			1. Area under curve<br>2. Time to peak	0.0131	*
			1. Area under curve<br>2. Response persistence	0.001	**

			1. Time to peak<br>2. Response persistence	0.0002	***
S2H	R48F03	Spearman correlation (non-parametric)	3. Peak<br>4. Percent responding cells	<0.0001	****
			3. Peak<br>4. Area under curve	<0.0001	****
			3. Peak<br>4. Time to peak	0.0391	*
			3. Peak<br>4. Response persistence	0.0391	*
			3. Percent responding cells<br>4. Area under curve	0.0014	*
			3. Percent responding cells<br>4. Time to peak	0.137	N.S.
			3. Percent responding cells<br>4. Response persistence	0.116	N.S.
			3. Area under curve<br>4. Time to peak	0.002	*
			3. Area under curve<br>4. Response persistence	0.002	*
			3. Time to peak<br>4. Response persistence	<0.0001	****
S3B	Peak Response ΔF/F	Two-Way ordinary ANOVA	Interaction	0.002	**
			Main effect of odor	<0.0001	****
			Main effect of compartment (somata Vs axons)	0.0006	***
		Sidak's multiple comparison test	ACV somata Vs axons	0.0002	***
			Ethyl acetate somata Vs axons	0.366	N.S.
			Ethyl lactate somata Vs axons	0.999	N.S.
			Geranyl acetate somata Vs axons	0.199	N.S.
			2-butanone somata Vs axons	>0.999	N.S.
			MCH somata Vs axons	0.999	N.S.
			Hexyl acetate somata Vs axons	0.958	N.S.
			2-heptanone somata Vs axons	0.996	N.S.
S3C	Peak responses ΔF/F	Pearson correlation	Somata Vs Axons	0.0158	*
S3D	Time to peak (s)	Two-Way ordinary ANOVA	Interaction	0.04	*
			Main effect of odor	<0.0001	****
			Main effect of compartment (somata Vs axons)	<0.0001	****
		Sidak's multiple comparison test	ACV somata Vs axons	0.805	N.S.
			Ethyl acetate somata Vs axons	0.367	N.S.
			Ethyl lactate somata Vs axons	<0.0001	****

			Geranyl acetate somata Vs axons	0.691	N.S.
			2-butanone somata Vs axons	<0.0001	N.S.
			MCH somata Vs axons	0.934	N.S.
			Hexyl acetate somata Vs axons	0.478	N.S.
			2-heptanone somata Vs axons	0.656	N.S.
S3E	Time to peak (s)	Pearson correlation	Somata Vs Axons	0.008	**
S3F	Responses persistence	Two-Way ordinary ANOVA	Interaction	0.082	N.S.
			Main effect of odor	<0.0001	****
			Main effect of compartment (somata Vs axons)	<0.0001	****
		Sidak's multiple comparison test	ACV somata Vs axons	0.995	N.S.
			Ethyl acetate somata Vs axons	0.991	N.S.
			Ethyl lactate somata Vs axons	<0.0001	****
			Geranyl acetate somata Vs axons	0.009	**
			2-butanone somata Vs axons	0.985	N.S.
			MCH somata Vs axons	0.998	N.S.
			Hexyl acetate somata Vs axons	0.009	**
			2-heptanone somata Vs axons	0.768	N.S.
S3G	Responses persistence	Pearson correlation	Somata Vs Axons	0.026	*
S4A	Walking speed	One-way non parametric ANOVA - Kruskal's-Wallis test	1. R37G11-GAL4<br>2. UAS- Shi^{ts1}<br>3. R37G11-GAL4> UAS- Shi^{ts1} 23°<br>4. R37G11-GAL4> UAS- Shi^{ts1} 32°	0.0185	*
		Dunn's multiple comparison test	1. R37G11-GAL4<br>2. UAS- Shi^{ts1}	> 0.9999	N.S.
			1. R37G11-GAL4<br>2. R37G11-GAL4> UAS- Shi^{ts1} 32°	0.4444	N.S.
			1. R37G11-GAL4<br>2. R37G11-GAL4> UAS- Shi^{ts1} 23°	0.8976	N.S.
			1. UAS- Shi^{ts1}<br>2. R37G11-GAL4> UAS- Shi^{ts1} 32°	> 0.9999	N.S.
			1. UAS- Shi^{ts1}<br>2. R37G11-GAL4> UAS- Shi^{ts1} 23°	0.0680	N.S.
			1. R37G11-GAL4> UAS- Shi^{ts1} 23°<br>2. R37G11-GAL4> UAS- Shi^{ts1} 32°	0.0309	*
S4B	2-Butanone	One-way non parametric ANOVA– Kruskal's-Wallis test	1. LH989-GAL4<br>2. UAS-Shi^{ts1}<br>3. LH989-GAL4> UAS-Shi^{ts1} 23˚<br>4. LH989-GAL4> UAS-Shi^{ts1} 32°	<0.0001	****
		Dunn's multiple comparison test	1. LH989-GAL4<br>2. UAS-Shi^{ts1}	0.6059	N.S.
			1. LH989-GAL4<br>2. LH989-GAL4> UAS-Shi^{ts1} 23˚	>0.9999	N.S.
			1. UAS-Shi^{ts1}<br>2. LH989-GAL4> UAS-Shi^{ts1} 23°	>0.9999	N.S.

		1. LH989-GAL4<br>2. LH989-GAL4> UAS-Shi^{ts1} 32°	<0.0001	****
		1. UAS-Shi^{ts1}<br>2. LH989-GAL4> UAS-Shi^{ts1} 32°	<0.0001	****
		1. LH989-GAL4> UAS-Shi^{ts1} 23°<br>2. LH989-GAL4> UAS-Shi^{ts1} 32°	<0.0001	****
Ethyl acetate	One-way non parametric ANOVA–Kruskal's-Wallis test	1. LH989-GAL4<br>2. UAS-Shi^{ts1}<br>3. LH989-GAL4> UAS-Shi^{ts1} 23˚<br>4. LH989-GAL4> UAS-Shi^{ts1} 32°	<0.0001	****
	Dunn's multiple comparison test	1. LH989-GAL4<br>2. UAS-Shi^{ts1}	0.0762	N.S.
		1. LH989-GAL4<br>2. LH989-GAL4> UAS-Shi^{ts1} 23˚	0.5141	N.S.
		1. UAS-Shi^{ts1}<br>2. LH989-GAL4> UAS-Shi^{ts1} 23°	>0.9999	N.S.
		1. LH989-GAL4<br>2. LH989-GAL4> UAS-Shi^{ts1} 32°	0.0255	*
		1. UAS-Shi^{ts1}<br>2. LH989-GAL4> UAS-Shi^{ts1} 32°	<0.0001	****
		1. LH989-GAL4> UAS-Shi^{ts1} 23°<br>2. LH989-GAL4> UAS-Shi^{ts1} 32°	0.0004	***
ACV	One-way non parametric ANOVA–Kruskal's-Wallis test	1. LH989-GAL4<br>2. UAS-Shi^{ts1}<br>3. LH989-GAL4> UAS-Shi^{ts1} 23˚<br>4. LH989-GAL4> UAS-Shi^{ts1} 32°	<0.0001	****
	Dunn's multiple comparison test	1. LH989-GAL4<br>2. UAS-Shi^{ts1}	>0.9999	N.S.
		1. LH989-GAL4<br>2. LH989-GAL4> UAS-Shi^{ts1} 23˚	0.8478	N.S.
		1. UAS-Shi^{ts1}<br>2. LH989-GAL4> UAS-Shi^{ts1} 23°	>0.9999	N.S.
		1. LH989-GAL4<br>2. LH989-GAL4> UAS-Shi^{ts1} 32°	0.0029	**
		1. UAS-Shi^{ts1}<br>2. LH989-GAL4> UAS-Shi^{ts1} 32°	0.0004	***
		1. LH989-GAL4> UAS-Shi^{ts1} 23°<br>2. LH989-GAL4> UAS-Shi^{ts1} 32°	<0.0001	****
Geranyl acetate	One-way non parametric ANOVA–Kruskal's-Wallis test	1. LH989-GAL4<br>2. UAS-Shi^{ts1}<br>3. LH989-GAL4> UAS-Shi^{ts1} 23˚<br>4. LH989-GAL4> UAS-Shi^{ts1} 32°	<0.0001	****
	Dunn's multiple comparison test	1. LH989-GAL4<br>2. UAS-Shi^{ts1}	0.3429	N.S.
		1. LH989-GAL4<br>2. LH989-GAL4> UAS-Shi^{ts1} 23˚	0.0346	*
		1. UAS-Shi^{ts1}<br>2. LH989-GAL4> UAS-Shi^{ts1} 23°	>0.9999	N.S.
		1. LH989-GAL4	<0.0001	****

			2. LH989-GAL4> UAS-Shi^{ts1} 32°		
			1. UAS-Shi^{ts1}<br>2. LH989-GAL4> UAS-Shi^{ts1} 32°	<0.0001	****
			1. LH989-GAL4> UAS-Shi^{ts1} 23°<br>2. LH989-GAL4> UAS-Shi^{ts1} 32°	<0.0001	****
	Hexyl acetate	One-way non parametric ANOVA–Kruskal's-Wallis test	1. LH989-GAL4<br>2. UAS-Shi^{ts1}<br>3. LH989-GAL4> UAS-Shi^{ts1} 23˚<br>4. LH989-GAL4> UAS-Shi^{ts1} 32°	<0.0001	****
		Dunn's multiple comparison test	1. LH989-GAL4<br>2. UAS-Shi^{ts1}	>0.9999	N.S.
			1. LH989-GAL4<br>2. LH989-GAL4> UAS-Shi^{ts1} 23˚	>0.9999	N.S.
			1. UAS-Shi^{ts1}<br>2. LH989-GAL4> UAS-Shi^{ts1} 23°	0.4117	N.S.
			1. LH989-GAL4<br>2. LH989-GAL4> UAS-Shi^{ts1} 32°	<0.0001	****
			1. UAS-Shi^{ts1}<br>2. LH989-GAL4> UAS-Shi^{ts1} 32°	<0.0001	****
			1. LH989-GAL4> UAS-Shi^{ts1} 23°<br>2. LH989-GAL4> UAS-Shi^{ts1} 32°	<0.0001	****
S5	Learning index for Ethyl acetate 10-4	Mann Whitney Non-parametric t-test	1. UAS- Shi^{ts1} No shock<br>2. UAS- Shi^{ts1} shock	<0.0001	****
	Learning index for ACV 10-4	Mann Whitney Non-parametric t-test	1. UAS- Shi^{ts1} No shock<br>2. UAS- Shi^{ts1} shock	0.02	*
S6	Time to peak	Two-Way Ordinary ANOVA	Interaction	0.56	N.S.
		Two-Way ordinary ANOVA<br>Sidak's multiple comparison test	Main effect of odor	<0.0001	****
			Main effect of condition (Fed Vs starved)	0.002	**
			Ethyl acetate fed Vs starved	>0.999	N.S.
			Ethyl lactate fed Vs starved	0.999	N.S.
			Geranyl acetate fed Vs starved	>0.999	N.S.
			2-butanone fed Vs starved	0.069	N.S.
			MCH fed Vs starved	0.999	N.S.
		Sidak's multiple comparison test	Hexyl acetate fed Vs starved	>0.999	N.S.
			2-heptanone fed Vs starved	0.995	N.S.
			Ethyl propionate fed Vs starved	0.914	N.S.
			Linalool fed Vs starved	0.999	N.S.
			Acetic acid fed Vs starved	0.009	**

S7B Top	avoidance index for 2-butanone Vs ethyl lactate	One-way non parametric ANOVA - Kruskal's-Wallis test	1. R37G11-GAL4<br>2. UAS- Shi^{ts1}<br>3. R37G11-GAL4> UAS- Shi^{ts1} 23°<br>4. R37G11-GAL4> UAS- Shi^{ts1} 32°	<0.0001	****
		Dunn's multiple comparison test	1. R37G11-GAL4<br>2. UAS- Shi^{ts1}	0.1292	N.S.
			1. R37G11-GAL4<br>2. R37G11-GAL4> UAS- Shi^{ts1} 23°	0.7440	N.S.
			1. UAS- Shi^{ts1}<br>2. R37G11-GAL4> UAS- Shi^{ts1} 23°	>0.999	N.S.
			1. R37G11-GAL4<br>2. R37G11-GAL4> UAS- Shi^{ts1} 32°	< 0.0001	****
			1. UAS- Shi^{ts1}<br>2. R37G11-GAL4> UAS- Shi^{ts1} 32°	0.3345	N.S.
			1. R37G11-GAL4> UAS- Shi^{ts1} 23°<br>2. R37G11-GAL4> UAS- Shi^{ts1} 32°	0.0020	**
	avoidance index for ethyl lactate Vs geranyl acetate	One-way non parametric ANOVA - Kruskal's-Wallis test	1. R37G11-GAL4<br>2. UAS- Shi^{ts1}<br>3. R37G11-GAL4> UAS- Shi^{ts1} 23°<br>4. R37G11-GAL4> UAS- Shi^{ts1} 32°	0.2461	N.S.
S7B Bottom	Learning index for ethyl lactate Vs 2-butanone	One-way non parametric ANOVA - Kruskal's-Wallis test	1. R37G11-GAL4<br>2. UAS- Shi^{ts1}<br>3. R37G11-GAL4> UAS- Shi^{ts1} 23°<br>4. R37G11-GAL4> UAS- Shi^{ts1} 32°	<0.0001	****
		Dunn's multiple comparison test	1. R37G11-GAL4<br>2. UAS- Shi^{ts1}	>0.999	N.S.
			1. R37G11-GAL4<br>2. R37G11-GAL4> UAS- Shi^{ts1} 23°	0.108	N.S.
			1. UAS- Shi^{ts1}<br>2. R37G11-GAL4> UAS-Shi^{ts1} 23°	0.753	N.S.
			1. R37G11-GAL4<br>2. R37G11-GAL4> UAS- Shi^{ts1} 32°	0.037	*
			1. UAS- Shi^{ts1}<br>2. R37G11-GAL4> UAS- Shi^{ts1} 32°	0.003	**
			1. R37G11-GAL4> UAS- Shi^{ts1} 23°<br>2. R37G11-GAL4> UAS- Shi^{ts1} 32°	<0.0001	****
	Learning index for geranyl acetate Vs ethyl lactate	One-way non parametric ANOVA - Kruskal's-Wallis test	1. R37G11-GAL4<br>2. UAS- Shi^{ts1}<br>3. R37G11-GAL4> UAS- Shi^{ts1} 23°<br>4. R37G11-GAL4> UAS- Shi^{ts1} 32°	0.144	N.S.
